# S-Adenosylmethionine Negatively Regulates the Mitochondrial Respiratory Chain Repressor MCJ in the Liver

**DOI:** 10.7150/ijbs.90104

**Published:** 2024-01-27

**Authors:** Lucía Barbier-Torres, Jyoti Chhimwal, So Yeon Kim, Komal Ramani, Aaron Robinson, Heping Yang, Jenny Van Eyk, Suthat Liangpunsakul, Ekihiro Seki, José M. Mato, Shelly C. Lu

**Affiliations:** 1 Karsh Division of Gastroenterology and Hepatology, Cedars-Sinai Medical Center, Los Angeles, CA, USA.; 2 Smidt Heart Institute and Advanced Clinical Biosystems Research Institute, Los Angeles, CA, USA.; 3 Division of Gastroenterology and Hepatology, Department of Medicine, Indiana University School of Medicine, Indianapolis, IN, USA.; 4 Department of Biochemistry and Molecular Biology, Indiana University School of Medicine, Indianapolis, IN, USA.; 5 Roudebush Veterans Administration Medical Center, Indianapolis, IN, USA;; 6 bioGUNE, Centro de Investigación Biomédica en Red de Enfermedades Hepáticas y Digestivas (Ciberehd), Basque Research and Technology Assembly (BRTA), Technology Park of Bizkaia, 48160 Derio, Bizkaia, Spain.

**Keywords:** MCJ, MATα1, S-adenosylmethionine, protein methylation, alcohol-associated liver disease, mitochondrial dysfunction

## Abstract

MCJ (Methylation-Controlled J protein), an endogenous repressor of the mitochondrial respiratory chain, is upregulated in multiple liver diseases but little is known about how it is regulated. S-adenosylmethionine (SAMe), the biological methyl donor, is frequently depleted in chronic liver diseases. Here, we show that SAMe negatively regulates MCJ in the liver. While deficiency in methionine adenosyltransferase alpha 1 (MATα1), enzyme that catalyzes SAMe biosynthesis, leads to hepatic MCJ upregulation, MAT1A overexpression and SAMe treatment reduced MCJ expression. We found that MCJ is methylated at lysine residues and that it interacts with MATα1 in liver mitochondria, likely to facilitate its methylation. Lastly, we observed that MCJ is upregulated in alcohol-associated liver disease, a condition characterized by reduced *MAT1A* expression and SAMe levels along with mitochondrial injury. MCJ silencing protected against alcohol-induced mitochondrial dysfunction and lipid accumulation. Our study demonstrates a new role of MATα1 and SAMe in reducing hepatic MCJ expression.

## Introduction

MCJ (Methylation-Controlled J protein), also known as DNAJC15, is a small protein (150 aa) and member of the HSP40 family of co-chaperones that acts as an endogenous negative regulator of mitochondrial respiration^1^. Unlike the other members of the family, MCJ is a transmembrane protein and is anchored to the mitochondrial inner membrane where it interacts with complex I of the electron transport chain inhibiting its activity^1^. Through this interaction, MCJ also interferes with the aggregation of mitochondrial supercomplexes, which maximize respiratory efficiency by facilitating the transport of electrons between the complexes and reducing its leak and production of reactive oxygen species (ROS)^1^. MCJ acts as a mitochondrial brake as its deficiency increases mitochondrial respiration and the production of ATP^1^. MCJ is expressed at higher levels in active metabolic tissues such as the heart, liver, and kidney but seems nonessential as *Mcj*-knockout (KO) mice show no phenotype^1^.

MCJ is upregulated in multiple liver diseases, including drug-induced liver injury (DILI)^2^, metabolic dysfunction associated steatotic liver disease (MASLD, previously known as non-alcoholic fatty liver disease)^3^, and cholestasis^4^, conditions characterized by mitochondrial injury. Its deficiency protected against these liver pathologies both in vitro and in vivo, as seen in *Mcj*-KO mice and wild type (WT) mice after *Mcj* silencing using specific siRNAs or Gal-NAc particles^3^. Since MCJ is a repressor of mitochondrial metabolism, its inhibition stimulates mitochondrial respiration, ATP production, and fatty acid oxidation, thus protecting against hepatotoxic insults such as acetaminophen^2^ and bile acids^4^, as well as against hepatic steatosis induced by fasting or high-fat diets^3^. Taken together, these findings demonstrate an important role for MCJ in the pathogenesis of liver diseases and validate it as a promising target for their treatment.

Although MCJ is emerging as a promising target for the treatment of a variety of liver diseases, the mechanism leading to its upregulation is not fully understood. MCJ is negatively regulated by CpG island methylation in ovarian cancer cells^5^,^6^. Similarly, lower methylation of *MCJ* promoter correlated with higher gene expression in livers of MASLD patients^3^. The only DNA methylation-independent mechanism of *MCJ* silencing was described in macrophages where IFNγ promotes the binding of the transcription factor Ikaros to the *MCJ* promoter in a casein kinase II-dependent manner to repress its expression^7^. How MCJ is regulated at the protein level remains to be investigated.

S-adenosylmethionine (SAMe) is the principal methyl donor and an important cellular antioxidant as it is a precursor of glutathione. The liver is the main organ responsible for its biosynthesis and normal hepatic SAMe levels are important for liver health and mitochondrial function. In chronic liver diseases SAMe levels fall, which has been associated to mitochondrial dysfunction. Here we examined whether MCJ is involved in mitochondrial dysfunction caused by low hepatic SAMe levels. We assessed MCJ expression in methionine adenosyltransferase 1a (*Mat1a*, gene responsible for SAMe biosynthesis in the liver)-KO mice and in alcohol-associated liver disease (ALD), as both exhibit mitochondrial injury^8^,^9^,^10^ and lower hepatic MAT1A expression and SAMe levels^11^,^12^. By using different in vitro and in vivo approaches, we confirmed that SAMe negatively regulates MCJ at the protein level in hepatocytes. In addition, we found that MCJ is methylated at lysine residues and that it interacts with MATα1 in liver mitochondria which may facilitate its methylation. Lastly, we identified MCJ as an important target for ALD. MCJ is upregulated in ALD and its silencing efficiently improved mitochondrial function and reduced lipid accumulation in vitro, supporting its therapeutic role for the treatment of the disease.

## Results

### MCJ is upregulated in the livers of *Mat1a*-KO mice

**Figure 1:**
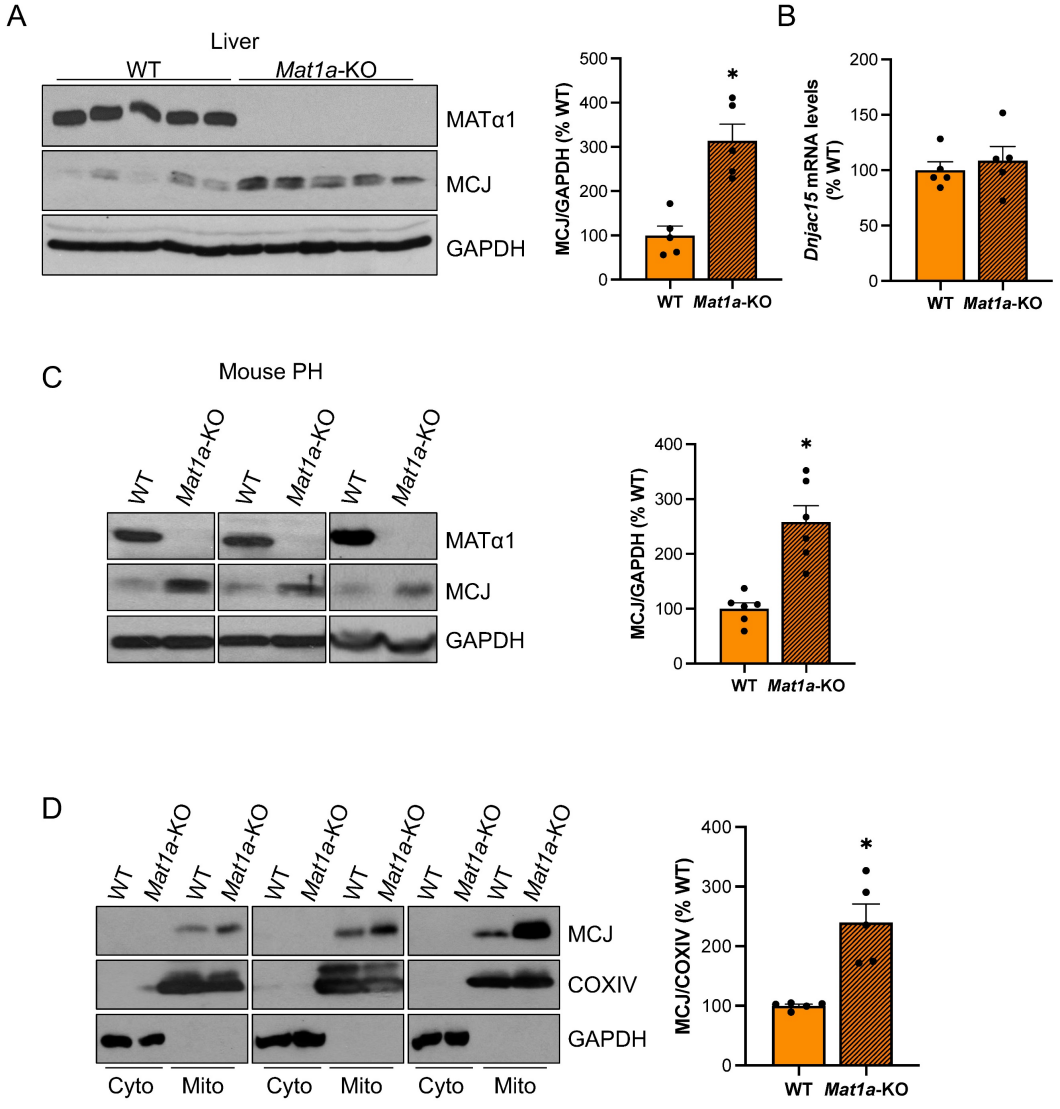
** MCJ protein levels are induced in the livers of *Mat1a*-KO mice**. (A) MATα1 and MCJ protein levels by western blotting and densitometric quantification in liver lysates from WT and *Mat1a*-KO mice. (B) *Dnajc15* expression measured by RT-PCR in livers from WT and *Mat1a*-KO mice. (C) MCJ protein levels by western blotting and densitometric quantification in primary hepatocytes (PH) from WT and *Mat1a*-KO mice. (D) MCJ protein levels by western blotting and densitometric quantification in cytosolic and mitochondrial fractions from WT and *Mat1a*-KO mouse livers. *P<0.05 *Mat1a*-KO vs WT.

Since MCJ is an endogenous inhibitor of mitochondrial respiration in the liver and *Mat1a*-KO mice are characterized by mitochondrial dysfunction, we examined whether MCJ expression was altered in this model. *Mat1a*-KO mice have chronically low hepatic SAMe levels and spontaneously develop non-alcoholic steatohepatitis (NASH)^13^ and hepatocellular carcinoma (HCC)^14^. We first evaluated MCJ protein levels in livers of 4-month-old WT and *Mat1a*-KO mice and found a 2.3-fold increase in the absence of *Mat1a* (Fig. 1A). Dnajc15 (gene that encodes MCJ) mRNA levels were unchanged (Fig. 1B), suggesting that MCJ upregulation occurs at the post-translational level. We also evaluated MCJ expression in younger mice and found a similar regulation (Figure S1A-B).

To ensure that MCJ overexpression in the livers of *Mat1a*-KO mice occurs in hepatocytes, we evaluated MCJ protein levels by western blotting in isolated primary hepatocytes. Fig. 1C shows that MCJ protein levels are highly increased in hepatocytes from *Mat1a*-KO mice compared to WT. It has been previously shown that hepatocytes express MCJ at a higher level compared to other cell types such as hepatic stellate cells and Kupffer cells^4^. We evaluated *DNAJC15* expression in human liver to a wider extent using an available public single-cell RNAseq dataset and found that while *MAT1A* is primarily expressed in hepatocytes, *DNAJC15* is ubiquitously expressed in the liver, with higher expression levels observed in hepatocytes, B cells, and lymphocytes (Figure S1C-D).

Lastly, we evaluated MCJ levels in the cytosolic and mitochondrial fractions from WT and *Mat1a*-KO livers. As expected, we did not detect any MCJ in the cytosol and found a significant upregulation in the mitochondrial content of MCJ in the absence of *Mat1a* (Fig. 1D). Since MATα1 (protein encoded by *MAT1A*) is also localized to the mitochondria of hepatocytes^8^,^10^, we also evaluated the possibility that MCJ could reciprocally regulate MATα1. For that, we assessed MATα1 protein levels in livers of WT and *Mcj-*KO mice. As shown in Fig. S2A, neither total nor mitochondrial MATα1 were altered in the absence of MCJ. Since *Mat1a*-KO mice have reduced levels of prohibitin 1 (PHB1), a well-known mitochondrial chaperone, we assessed MCJ expression in livers of *Phb1*-KO mice. MCJ protein and mRNA levels were unchanged in these mice (Fig. S2B-D), ruling out that PHB1 is regulating MCJ expression under MAT1A and SAMe deficiency.

### MAT1A negatively regulates MCJ expression in hepatocytes via SAMe

**Figure 2:**
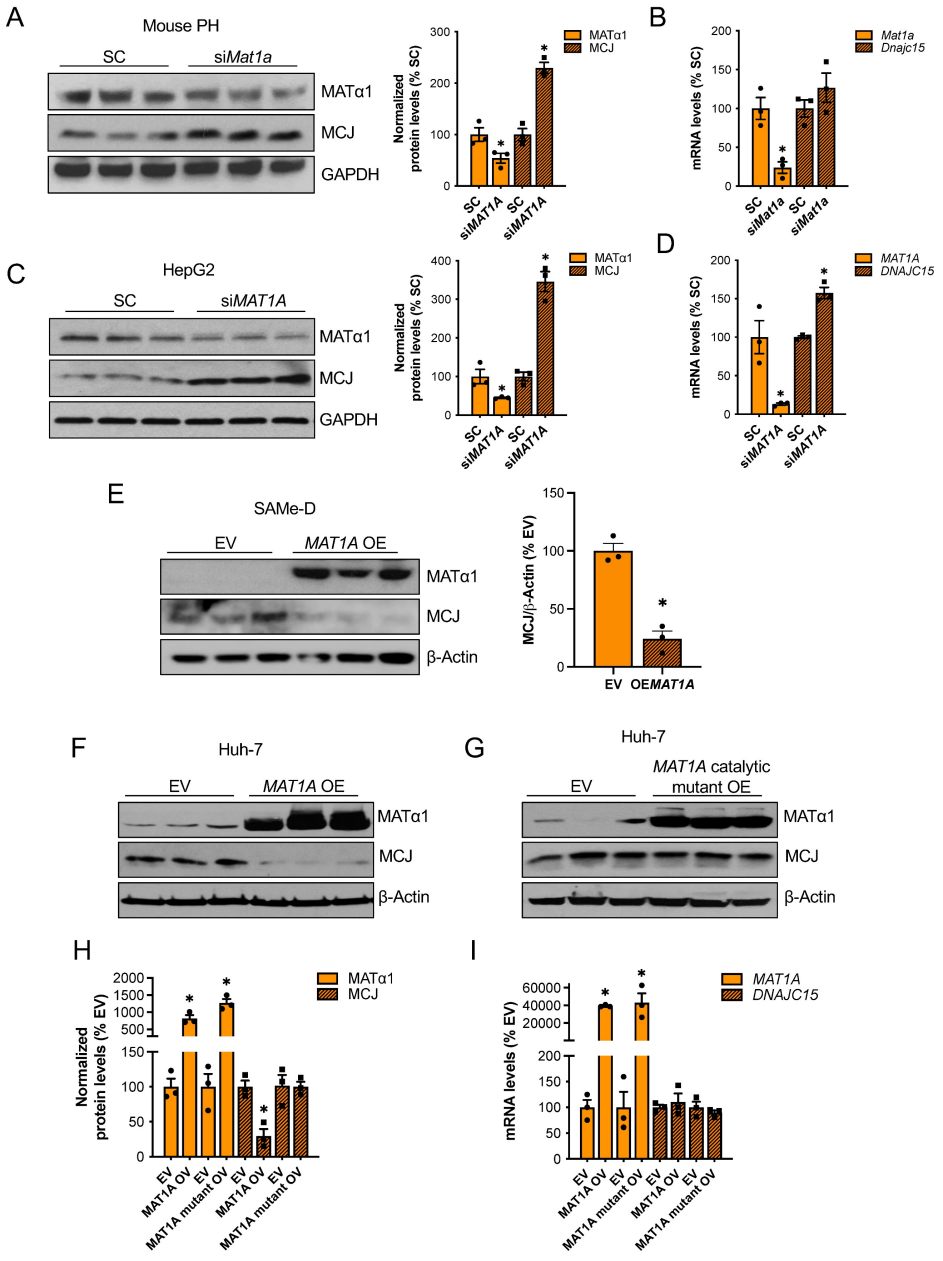
** MAT1A negatively regulates MCJ via SAMe**. (A) MCJ and MATα1 protein levels by western blotting and densitometric quantification and (B) *Dnajc15* and *Mat1a* expression measured by RT-PCR in mouse primary hepatocytes (PH) after *Mat1a* silencing. (C) MCJ and MATα1 protein levels by western blotting and densitometric quantification and (D) *DNAJC15* and *MAT1A* expression measured by RT-PCR in HepG2 cells after *MAT1A* silencing. (E) MCJ and MATα1 levels by western blotting and densitometric quantification (lower panel) after MAT1A overexpression in SAMe-D cells. (F-H) MCJ and MATα1 levels by western blotting in Huh-7 cells after (F) MAT1A WT and (G) the catalytic mutant overexpression and (H) densitometric quantification. (I) *DNAJC15* and *MAT1A* expression measured by RT-PCR in Huh-7 cells after *MAT1A* WT and catalytic mutant overexpression. *P<0.05 si*MAT1A* vs SC; MAT1A OV vs EV; MAT1A mutant OV vs EV.

MAT1A and SAMe chronic deficiency alters many pathways that lead to liver injury^15^. To study the effect of MAT1A on MCJ more specifically, we transiently altered MAT1A levels in different liver cells and evaluated its impact on MCJ expression. *Mat1a* knockdown in WT primary hepatocytes (Fig. 2A-B) and HepG2 cells (Fig. 2C-D) upregulated MCJ protein levels to 2.3-fold and 3.4-fold, respectively. Although *Dnajc15* mRNA levels were unaltered in *Mat1a-*KO mice (Fig. 1B), acute silencing in mouse hepatocytes and HepG2 cells increased Dnajc15/DNAJC15 expression by 20-50%, respectively (Fig. 2C-D), suggesting that MCJ is also regulated at the transcriptional level. However, the magnitude of increase at the MCJ protein level (2.3 - 3.4-fold of control) was much higher than the mRNA level (20 - 50% higher), which indicates that the regulation is mainly at the post-translational level in both mouse hepatocytes and HepG2 cells.

We next examined if MAT1A negatively regulates MCJ in two cell lines with absent or very low MAT1A levels: SAMe-D cells -cell line isolated from *Mat1a*-KO HCC^16^- and Huh-7 cells. *MAT1A* overexpression strongly reduced MCJ protein levels in SAMe-D cells (Fig. 2E) and Huh-7 cells (Fig. 2F). To evaluate whether the MATα1 regulates MCJ protein levels through SAMe, we overexpressed a MATα1 catalytic mutant in Huh-7 cells. We found that SAMe mediates MCJ downregulation as the MATα1 catalytic mutant did not reduce MCJ levels (Fig. 2G and H). *DNAJC15* mRNA levels remained unchanged in Huh-7 cells after MAT1A overexpression (Fig. 2I), suggesting that MCJ regulation by MAT1A in these cells is strictly post-translational. Altogether, these results demonstrate that MATα1 negatively regulates MCJ in hepatocytes at the post-translational level via SAMe.

**Figure 3:**
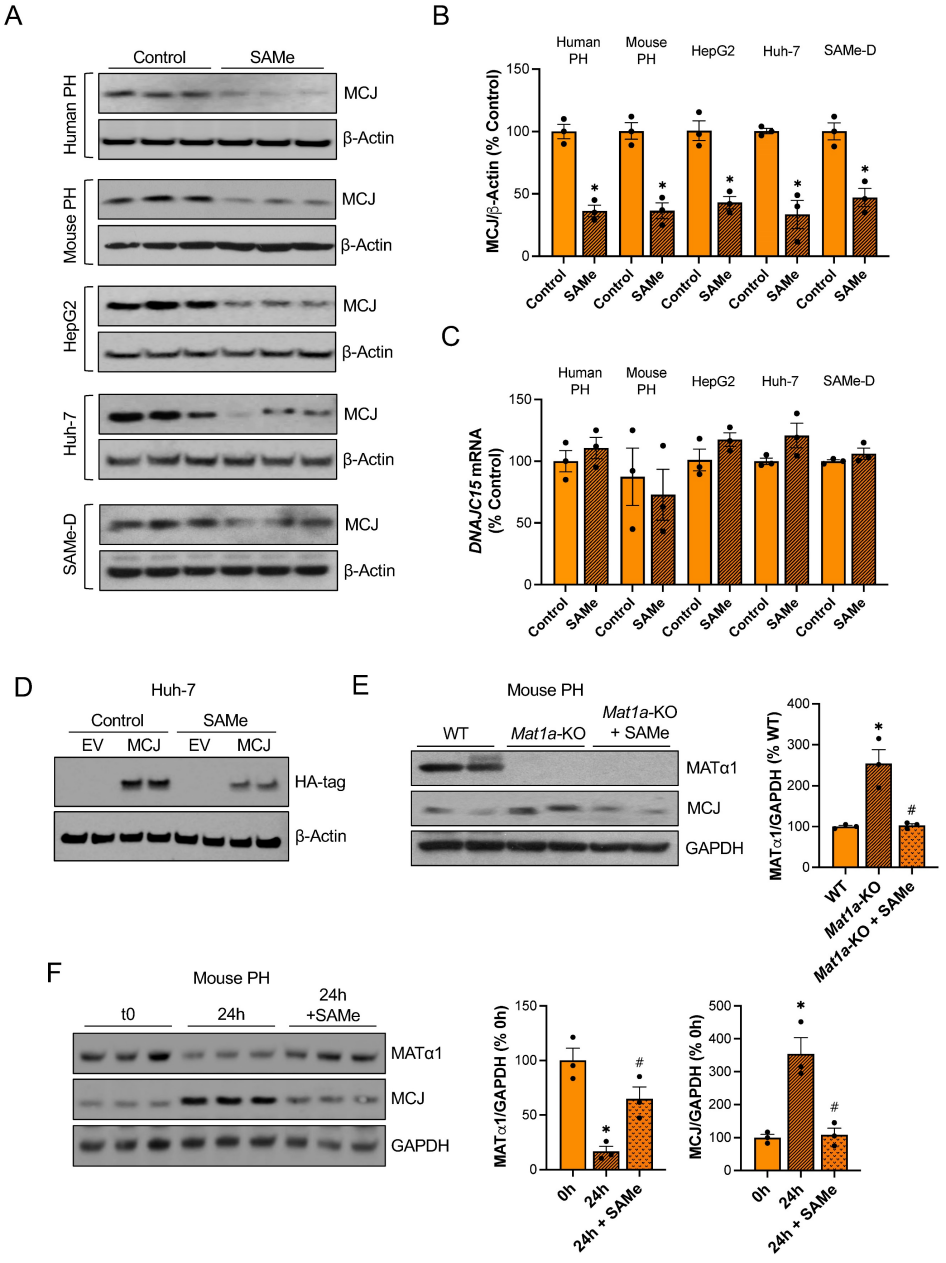
** SAMe reduces MCJ protein levels in hepatocytes.** (A) MCJ protein levels by western blotting, (B) quantification and (C) *DNAJC15* mRNA levels in human primary hepatocytes (PH), mouse PH, HepG2, Huh-7 and SAMe-D cells after 24 hours of SAMe treatment. (D) MCJ-HA levels measured by western blotting in Huh-7 cells after MCJ overexpression for 48 hours and 24 hours of SAMe treatment. (E) MCJ and MATα1 levels by western blotting and densitometric quantification in WT, *Mat1a*-KO and *Mat1a*-KO PH treated with SAMe for 24 hours. (F) MCJ and MATα1 protein levels by western blotting and densitometric quantification in mouse PHs after isolation (0h) and cultured for 24 hours with or without SAMe. *P<0.05 SAMe vs Control; *Mat1a*-KO vs WT; 24 hours vs 0 hours and ^#^P<0.05 *Mat1a*-KO + SAMe vs *Mat1a*-KO; 24 hours + SAMe vs 24 hours.

To evaluate if SAMe directly regulates MCJ in the liver, we treated different liver cells with SAMe in vitro. Since primary hepatocytes dedifferentiate in collagen-coated plates and MATα1 and SAMe levels fall during hepatocyte dedifferentiation^17^, we used matrigel to keep both human and mouse primary hepatocytes differentiated while treating them with SAMe 1 mM for 24 hours. As shown in Fig. 3A-B, SAMe reduced MCJ protein levels by 60% while not changing *DNAJC15* mRNA levels (Fig. 3C). Similarly, 24 hours of 500 µM SAMe treatment (500 µM SAMe was used for cancer cells since 1 mM may induce cell death^18^) significantly reduced MCJ protein levels in HepG2, Huh-7 and SAMe-D cells (Fig. 3A-B) without altering MCJ at the mRNA level (Fig. 3C). SAMe exerted a time- and dose-dependent effect on lowering MCJ expression in these cell lines (Fig. S3A-B). SAMe at 500 µM showed the maximum effect on MCJ under these conditions while showing no effect on cell viability (Fig. S3C). MCJ is expressed ubiquitously and SAMe is present in all mammalian cells so we evaluated whether this regulation also occurs in other cell types. We treated RAW 264.7 (murine macrophages), RKO and HT-29 (colon cancer), LX-2 (hepatic stellate cells), and MCF-7 (breast cancer) cells with SAMe 500 µM for 24 hours and assessed MCJ levels by western blot. As shown in Figure S4, we observed a similar drop in MCJ expression in all of them.

In order to discard the possibility that methylation could be interfering with MCJ epitope-antibody recognition, we also evaluated the effect of SAMe on exogenous MCJ that carries a HA-tag in the N-terminal. We observed that overexpressed MCJ was also downregulated by SAMe treatment (Fig. 3D).

Next, to test our hypothesis that MCJ upregulation in *Mat1a*-KO livers is caused by SAMe deficiency, we evaluated whether SAMe treatment could normalize MCJ protein expression in *Mat1a*-KO primary hepatocytes. As seen before, we found a 2.5-fold increase in MCJ protein levels in *Mat1a*-KO hepatocytes (Fig. 1C) and observed that SAMe treatment normalized it to WT levels (Fig. 3E). We then investigated if SAMe was also able to block MCJ upregulation during hepatocyte dedifferentiation. For that, we cultured WT primary hepatocytes with or without SAMe 1 mM for 24 hours and evaluated MATα1 and MCJ protein expression. SAMe treatment effectively maintained higher MATα1 levels and inhibited MCJ upregulation (Fig. 3F).

Since SAMe and MAT1A deficiency can lead to mitochondrial changes^8^,^10^,^19^,^20^, we evaluated whether SAMe treatment could have affected the overall content of mitochondria in our experiments. Using a cocktail antibody that detects different oxidative phosphorylation (OXPHOS) system subunits of the electron transport chain, we evaluated the mitochondrial content in livers of WT mice treated with SAMe, and Huh-7 and HepG2 cells treated with SAMe. Except for complex II in WT livers, which was upregulated, SAMe treatment did not change the levels of these subunits, indicating that, under these conditions, SAMe did not alter mitochondrial content (**Figure S5A**). This finding suggests that SAMe's effect on MCJ is specific and not due to global mitochondrial changes.

Since SAMe has been associated with mRNA translation^21^, we evaluated whether SAMe could be affecting MCJ translation. We assessed *MCJ* mRNA levels in polysomes isolated from Huh-7 cells treated with SAMe but did not observe any changes (Fig. S5B). Taken together, these data demonstrate that SAMe is a strong inhibitor of MCJ acting primarily at the post-translational level.

### MATα1 and MCJ interact in liver mitochondria

**Figure 4:**
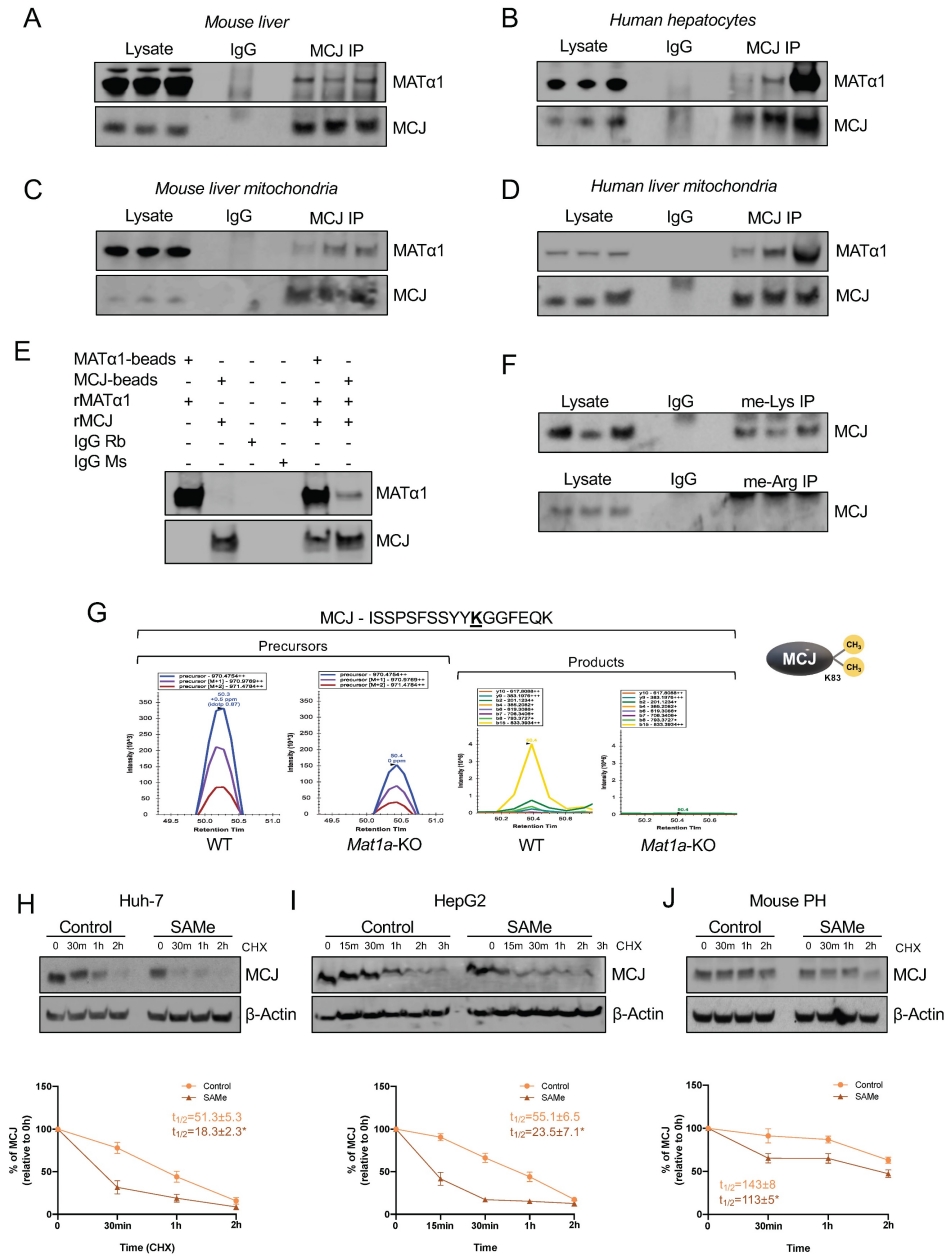
** MATα1 and MCJ interact in liver mitochondria.** (A) Immunoprecipitation (IP) analyses using MATα1 antibody in mouse liver lysates and (B) human hepatocyte lysates. (C) IP analyses using MCJ (left panel) and MATα1 antibodies (right panel) in mouse liver mitochondrial lysates. (D) IP analyses using MATα1 antibody in human liver mitochondrial lysates. (E) IP analysis using MATα1 and MCJ recombinant proteins and beads conjugated to anti- MATα1 and anti-MCJ antibodies. IgG was used as a negative control. (F) IP analyses in mouse liver lysates using antibodies against methyl-lysine and methyl-arginine. (G) Extracted ion chromatograms (XIC) for a doubly charged peptide indicative of MCJ K83 di-methylation. ISSPSFSSYYKGGFEQK has a co-eluting precursor trace with site specific product ions for MCJ K83 di-methylation and is differentially expressed between WT and *Mat1a*-KO. (H-J) MCJ levels by western blotting (upper panels) and quantification (lower panels) in (H) Huh-7, (I) HepG2 cells and (J) mouse PHs treated with SAMe for 24 hours before cycloheximide (CHX) addition. Graphs show MCJ's t_1/2_ with and without SAMe treatment. *P<0.05 SAMe vs Control.

Based on our previous findings that the interaction of MATα1 with Cytochrome P450 2E1 (CYP2E1) was associated with higher methylated but lower levels of mitochondrial CYP2E1^8^, we evaluated whether MATα1 and MCJ interact. Co-immunoprecipitation (IP) analyses in mouse liver (Fig. 4A) and human hepatocyte lysates (Fig. 4B) demonstrated that these proteins interact in the liver. Additionally, co-IP analyses in mouse (Fig. 4C) and human (Fig. 4D) liver mitochondrial fractions confirmed that these proteins interact within the organelle. Lastly, to determine whether MCJ and MATα1 interact with each other directly, we also performed IP analysis using recombinant proteins and antibodies conjugated to beads and as shown in Fig. 4E, we found that these two proteins can interact directly. Altogether, these results show that MATα1 interacts with MCJ in hepatic mitochondria and suggest that the enzyme could be also involved in the methylation of MCJ.

### SAMe negatively regulates MCJ's stability

All known mechanisms describing MCJ regulation are at the transcriptional level and lead to its silencing^5^,^6^,^7^. DNA methylation negatively regulates *MCJ* mRNA levels in various tissues^5^,^6^ but whether methylation also occurs at the protein level has never been explored. SAMe negatively regulates MCJ protein levels, suggesting that methylation might regulate MCJ protein stability. Using GPS-MSP, Malab and PSSME methylation prediction softwares, we identified 5 highly scored potential lysine methylation sites (K72, K83, K89, K107, and K125) and 4 arginine methylation sites (R2, R33, R53 and R92) on the MCJ protein sequence (Fig. S6A). To investigate whether MCJ is methylated, we first performed co-IP analyses in mouse liver lysates using specific methyl-lysine and methyl-arginine antibodies followed by western blotting against MCJ. As shown in Fig. 4F we found that hepatic MCJ is methylated at lysine residues. Although methylation at arginine residues was also predicted, co-IP analyses were negative (Fig. 4F) so no further experiments were performed. We next performed MCJ co-IP followed by mass spectrometry (MS) analyses in WT and *Mat1a*-KO liver lysates to identify methylated residues. Using this approach, we confirmed that MCJ is di-methylated at lysine 83 (Fig. 4G and Fig. S6B). Interestingly, we found that MCJ methylation at this residue is higher in WT livers compared to *Mat1a*-KO (Fig. 4G), which correlates with higher SAMe levels and lower MCJ protein expression. To study the effect of methylation at lysine residues on MCJ protein stability, we generated MCJ mutants that can't be methylated (lysine > alanine) at residues 72, 83, 89, 107 and 125. We expressed MCJ WT along with all the mutants (K72A, K83A, K89A, K107A and K125A) in Huh-7 cells that were treated with SAMe 500 µM for 24 hours and assessed MCJ protein levels by western blotting. However, we did not find that any of the mutations protected MCJ from SAMe-induced downregulation as compared to MCJ WT (Fig. S6C). We also assessed the effect of SAMe on MCJ half-life using cycloheximide chase assay in different cells and observed that SAMe significantly reduced MCJ's stability (Fig. 4H-J). MCJ's half-life was reduced by more than half in both Huh-7 (Fig. 4H) and HepG2 (Fig. 4I) cells and by 30% in mouse primary hepatocytes (Fig. 4J). Taken together, these data show that SAMe significantly destabilizes MCJ protein.

**Figure 5:**
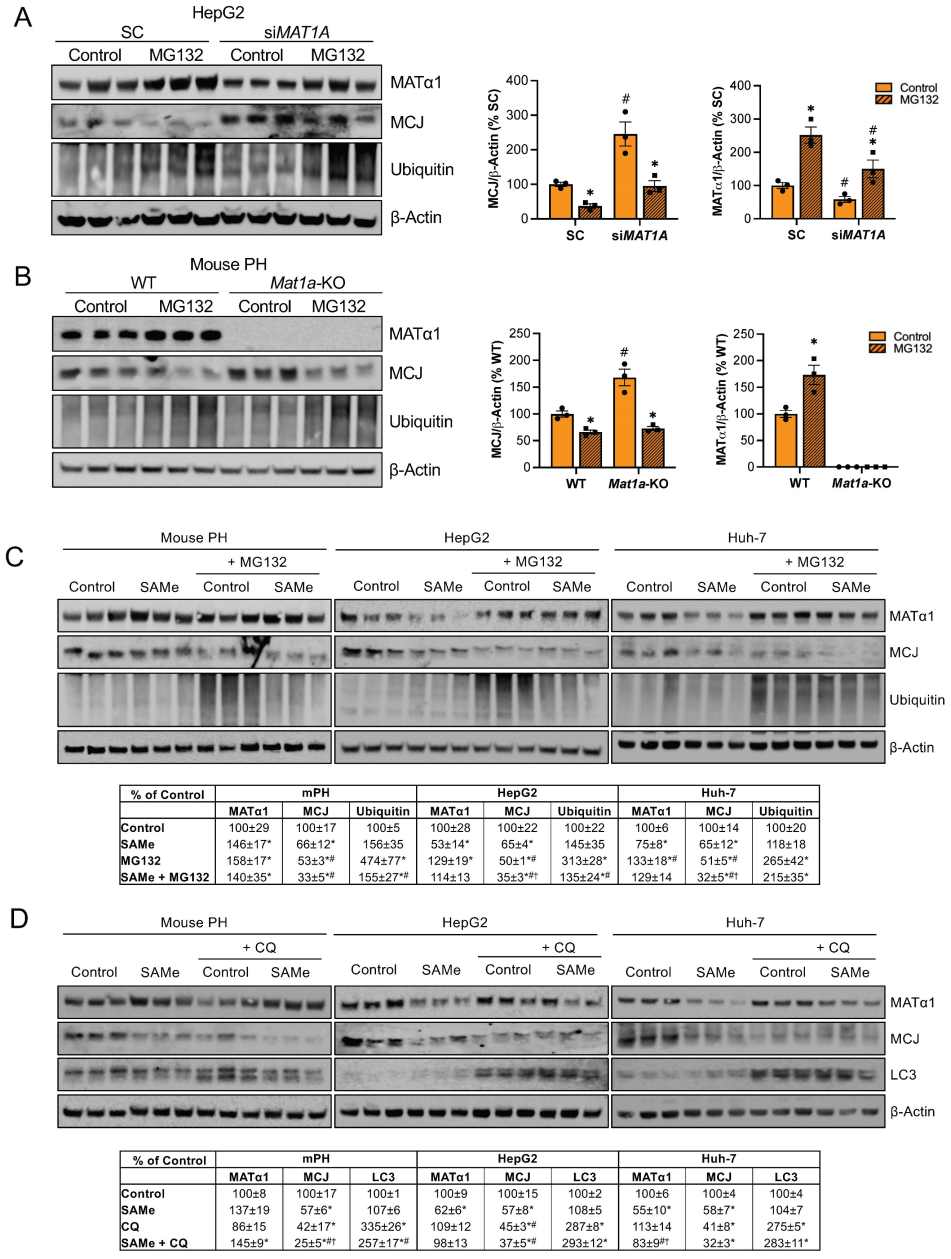
** SAMe does not regulate MCJ via proteasomal or autophagosomal degradation.** (A) MCJ and MATα1 levels by western blotting and quantification in HepG2 cells after *MAT1A* silencing for 48 hours and treatment with the proteasome inhibitor MG132 for the last 6 hours. (B) MCJ and MATα1 levels by western blotting and quantification in PH from WT and *Mat1a*-KO mice treated with the proteasome inhibitor MG132 for 6 hours. (C) MCJ, MATα1 and protein ubiquitination levels by western blotting and quantification (table below) in mouse PH, HepG2, and Huh-7 cells after treatment with SAMe for 24 hours and the proteasome inhibitor MG132 (20μM) for the last 6 hours. (D) MCJ, MATα1 and LC3 levels by western blotting and quantification (table below) in mouse PH, HepG2, and Huh-7 cells after treatment with SAMe for 24 hours and the autophagy inhibitor chloroquine (CQ) (50μM) for the last 6 hours. *P<0.05 MG132 vs Control; SAMe vs Control; MG132 or CQ vs Control; SAMe+MG132 or SAMe+CQ vs Control; ^#^P<0.05 si*MAT1A* vs SC; *Mat1a*-KO vs WT; MG132 or CQ vs SAMe; SAMe+MG132 or SAMe+CQ vs SAMe. ^†^P<0.05 SAMe+MG132 or SAMe+CQ vs MG132 or CQ.

How MCJ is degraded has never been explored. Mitochondrial proteins can be degraded by different mechanisms including proteasomal degradation, mitophagy and mitochondrial proteolysis^22^. The degradation of nuclear-encoded mitochondrial proteins via the proteasome has been already described^23^ and we have previously reported that methylation of mitochondrial CYP2E1 leads to its degradation through this pathway^8^. To investigate if MATα1 downregulates MCJ by inducing its proteasomal degradation, we first silenced *MAT1A* and treated HepG2 cells with the inhibitor MG132 10 µM for 6 hours. As shown in Figure 5A, MAT1A silencing increased MCJ expression levels, and this effect remained after treatment with MG132. Blocking the proteasomal pathway not only did not lead to MCJ accumulation but further reduced its expression. To examine if this was caused by an increase in MATα1 content, we treated *Mat1a*-KO hepatocytes with MG132 (Figure 5B). We found that MG132 reduced MCJ protein levels in hepatocytes lacking MAT1A to the level of WT, suggesting that the negative effect of MG132 on MCJ is independent of MAT1A.

We also examined the effect of MG132 after SAMe treatment (SAMe for 24 hours along with MG132 for the last 6 hours) in HepG2, Huh-7 and mouse primary hepatocytes and obtained the same result (Fig. 5C). Inhibiting the proteasomal activity reduced MCJ expression, concluding that MCJ is not degraded via the proteasome. SAMe co-treatment with MG132 in HepG2 and Huh-7 cells further reduced MCJ level (also lower in mouse hepatocytes but was not statistically significant) (Fig. 5C), which indicates SAMe's effect is different from that of MG132. We next evaluated whether MCJ could be degraded via autophagy/mitophagy. For that, we co-treated HepG2, Huh-7, and mouse primary hepatocytes with the inhibitor chloroquine (CQ) for 6 hours during a 24-hour SAMe treatment. As shown in Fig. 5D, MCJ protein levels were lower when autophagy was blocked, which excluded this molecular pathway as the responsible for SAMe-induced MCJ degradation. Since MATα1 protein levels were unaltered after CQ treatment, it is likely that the negative effect of CQ on MCJ is independent of SAMe as well. SAMe addition did not alter autophagy and at least in mouse hepatocytes, further reduced MCJ level indicating they work via distinct mechanisms.

### MCJ contributes to mitochondrial dysfunction induced by MAT1A deficiency

**Figure 6:**
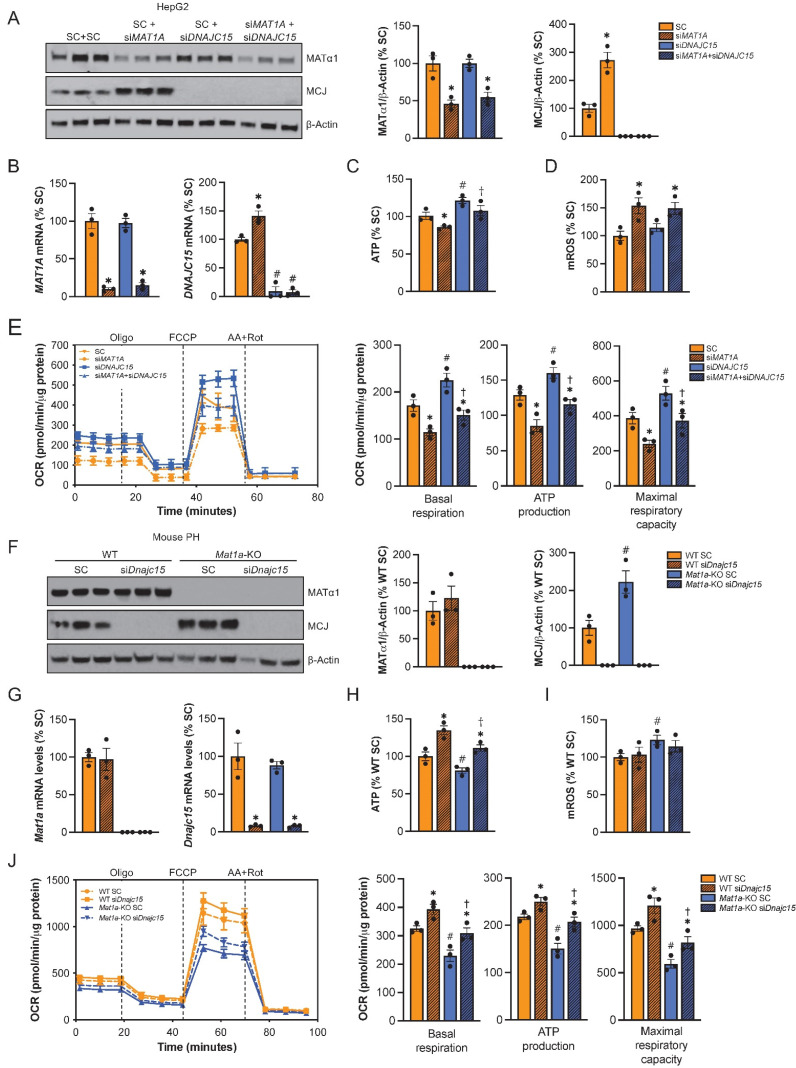
**MCJ contributes to mitochondrial dysfunction caused by MAT1A deficiency.** (A) MCJ and MATα1 levels by western blotting and densitometric quantification, (B) *DNAJC15* and *MAT1A* mRNA levels by RT-PCR, (C) ATP levels, (D) mitochondrial ROS (mROS) production, (E) mitochondrial respiration (left panel) and basal respiration, ATP production and maximal respiratory capacity (right panel) using the Seahorse analyzer in HepG2 cells after *MAT1A, DNAJC15* or* MAT1A+DNAJC15* silencing for 48 hours. *P<0.01 si*MAT1A* vs SC; si*MAT1A*+si*DNAJC15* vs si*DNAJC15.* ^#^P<0.01 si*DNAJC15* vs SC; si*MAT1A*+si*DNAJC15* vs SC*.* ^†^P<0.05 si*MAT1A*+si*DNAJC15* vs si*MAT1A.* (F) MCJ and MATα1 levels by western blotting and densitometric quantification, (G) *Dnajc15* and *Mat1a* mRNA levels by RT-PCR, (H) ATP levels, (I) mitochondrial ROS (mROS) production, (J) mitochondrial respiration (left panel) and basal respiration, ATP production and maximal respiratory capacity (right panel) using the Seahorse analyzer in primary hepatocytes (PH) from WT and *Mat1a*-KO mice cells after *Dnajc15* silencing for 36 hours. *P<0.01; si*Dnajc15* vs SC. ^#^P<0.01 *Mat1a*-KO SC vs WT SC. ^†^P<0.05 *Mat1a*-KO si*Dnajc15* vs WT si*Dnajc15.*

To evaluate the extent to which the effect of reduced MAT1A on mitochondrial activity is MCJ-dependent, we silenced MAT1A, MCJ, or both in HepG2 cells (Fig. 6A-B) and assessed multiple mitochondrial function markers. We observed that MAT1A deficiency had a negative effect on mitochondrial function in HepG2 as observed by reduced ATP levels (Fig. 6C), increased mitochondrial ROS (mROS) production (Fig. 6D) and reduced mitochondrial respiration (Fig. 6E).

While basal respiration, maximal respiratory capacity and mitochondria-dependent production of ATP were all reduced in the si*MAT1A* group, MCJ silencing raised these parameters (Fig. 6E right panel). MAT1A and MCJ double silencing showed same levels as the control with the exception of mROS, which remained unaltered (Fig. 6C-E), suggesting that MCJ silencing was able to compensate for MAT1A-deficiency induced mitochondrial dysfunction.

We also evaluated the effect of MCJ on the mitochondrial function of *Mat1a*-KO hepatocytes (Fig. 6F-J). For that, we silenced *Dnajc15* in both WT and *Mat1a*-KO primary hepatocytes (Fig. 6F) and assessed mitochondrial activity as we did for HepG2 cells (Fig. 6G-J). MAT1A deficiency had a negative effect on mitochondrial respiration as observed by lower ATP levels (Fig. 6H), higher mROS (Fig. 6I), and reduced oxygen consumption rate (Fig. 6J). Basal respiration, mitochondrial ATP production and maximal respiratory capacity were found reduced in *Mat1a*-KO hepatocytes (Fig. 6J right panel). *Dnajc15* silencing significantly increased mitochondrial respiration and ATP production without having any impact on mROS production in both WT and *Mat1a*-KO cells. The fact that mitochondrial respiration in *Mat1a*-KO after *siDnajc15* was comparable to that one of WT suggests that MCJ is an important mediator of mitochondrial dysfunction in the *Mat1a*-KO mice.

Altogether, these findings support that MCJ upregulation is another mechanism by which reduced SAMe levels promote mitochondrial impairment in the liver.

### MCJ is a target for alcohol-associated liver disease

**Figure 7:**
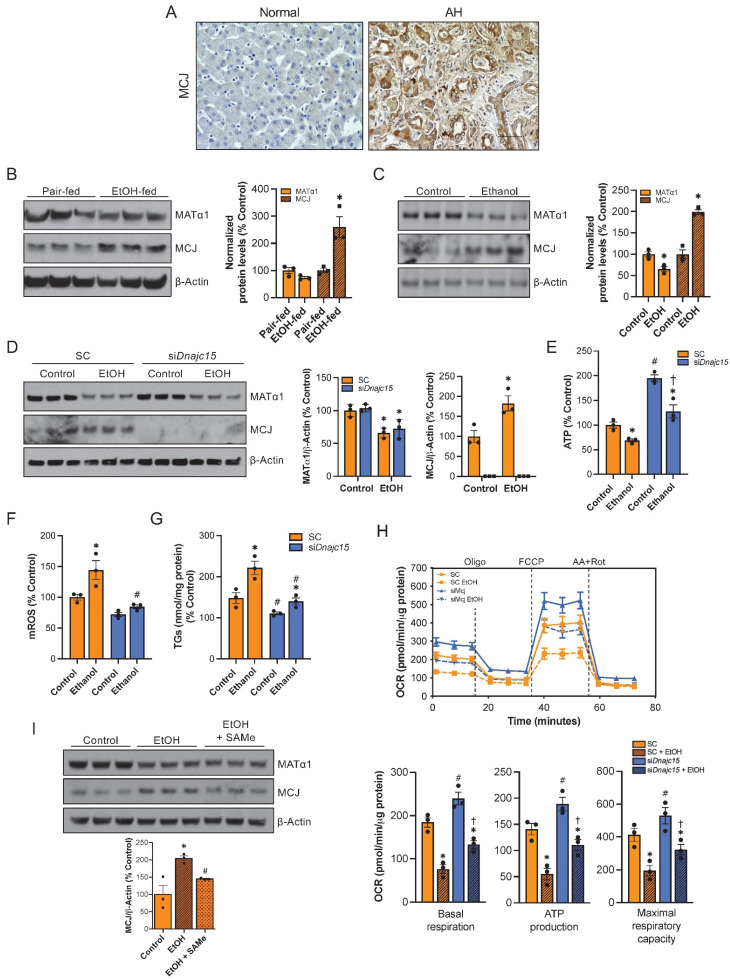
** MCJ is a therapeutic target for ALD.** (A) MCJ levels by immunohistochemistry in normal and alcoholic hepatitis (AH) human liver samples. (B) MCJ and MATα1 levels by western blotting and densitometric quantification in livers from pair-fed and ethanol-fed mice following the NIAAA model. (C) MCJ and MATα1 levels by western blotting and densitometric quantification in AML-12 cells treated with ethanol (100mM) for 48 hours. (D) MCJ and MATα1 levels by western blotting and densitometric quantification in AML-12 cells after 48 hours of *Dnajc15* silencing and ethanol treatment. (E) ATP levels, (F) mitochondrial ROS (mROS), (G) triglycerides (TGs) levels, (H) mitochondrial respiration (left panel) and basal respiration, ATP production and maximal respiratory capacity (right panel) using the Seahorse analyzer in AML-12 cells after 48 hours of *Dnajc15* silencing and ethanol treatment. (I) MCJ and MATα1 levels by western blotting and densitometric quantification in AML-12 cells treated with ethanol (100 mM) or ethanol + SAMe (500 µM) for 48 hours. *P<0.05 AH vs Normal; ethanol-fed vs pair-fed; ethanol vs control. ^#^P<0.05 s*iDnajc15* vs SC; EtOH + SAMe vs EtOH.

Since reduced levels of SAMe and MAT1A along with mitochondrial dysfunction occur frequently in ALD^9^,^10^,^11^, we explored whether MCJ could play a role in its pathogenesis. First, we measured MCJ expression by immunohistochemistry in normal and alcoholic hepatitis (AH) human liver samples. AH is a severe form of ALD characterized by hepatic inflammation^24^. As shown in Figure 7A and S7A, we found higher MCJ protein levels in human ALD. We next assessed MCJ levels in livers of mice subjected to the NIAAA model, which consists of a 10-day alcohol feeding followed by a single binge^25^. Similarly, we found a negative correlation between MCJ and MATα1. In this case, we observed a 1.7-fold increase in MCJ protein levels and lower MATα1 (Fig. 7B) but did not find any changes at the mRNA level (Fig. S7B). Lastly, the mouse hepatocyte cell line alpha mouse liver 12 (AML-12) treated with ethanol (100 mM for 48 hours) recapitulated human AH and in vivo findings. Ethanol reduced MATα1 total protein levels by 35% and increased MCJ protein level by 100% (Fig. 7C). We observed a non-significant reduction in mRNA levels in AML-12 cells treated with ethanol (Fig. S7C). It should be noted that we observed a reduction on MCJ protein levels in AML-12 cells treated with ethanol for 24 hours correlating with unaltered MATα1 levels but by 48 hours MCJ protein levels were clearly induced, as MATα1 levels dropped (Figure S7D). We next studied if MCJ induction plays a role in the pathogenesis of ALD. For that, we silenced *Dnajc15* using a specific siRNA in AML-12 cells (Fig. 7D) and evaluated the effect of alcohol on mitochondrial function and triglyceride accumulation. *Dnajc15* silencing attenuated ethanol-induced ATP depletion (Fig. 7E), mROS production (Fig. 7F) and accumulation of triglycerides (Fig. 7G). Mitochondrial respiration experiments using the Seahorse analyzer confirmed that lowering MCJ expression increases basal respiration, mitochondrial-dependent ATP production and maximal respiratory capacity in hepatocytes and that its silencing attenuated ethanol-induced mitochondrial respiration impairment (Fig. 7H). Lastly, supporting the negative effect that SAMe has on MCJ expression levels, we observed that SAMe co-treatment, which is known to be beneficial in ALD, attenuated the induction of MCJ by ethanol in AML-12 cells (**Figure 7I**). Overall, we found that MCJ upregulation is a major contributor to ethanol-induced mitochondrial dysfunction and to the pathogenesis of ALD.

## Discussion

MCJ (DNAJC15) is a mitochondrial protein that belongs to the HSP40 family of co-chaperones^5^ but its function as a co-chaperone seems not essential^26^. It acts as a negative regulator of mitochondrial respiration, function that has been attributed to its unique N-terminal that faces the mitochondrial intermembrane space and interacts with complex I of the electron transport chain^1^. Through this interaction, MCJ inhibits the activity of complex I and the aggregation of mitochondrial supercomplexes, repressing respiration^1^.

MCJ owns it name (methylation-J controlled protein) to its discovery in chemoresistant ovarian cancer cells where it was silenced by CpG island methylation^5^. A more recent study has shown that MCJ methylation at the promoter level also leads to its silencing in breast cancer^27^. The only known mechanisms shown to regulate MCJ expression are at the transcriptional level. Although MCJ upregulation in liver diseases occurs predominantly at the protein level, no post-translational mechanism underlying its overexpression has been described to date. Similarly, how MCJ protein is degraded remains unknown. Here, we describe that SAMe negatively regulates MCJ protein in the liver. It is worth mentioning that MCJ is expressed at a higher level in hepatocytes although it is ubiquitously expressed in all liver cell types.

SAMe is the main donor of methyl groups in the cell and a key regulator of most biological pathways^15^. It is used for methylation of RNA, DNA, and proteins and is also a precursor of glutathione, which makes it an important antioxidant as well^15^. SAMe is present in all cells and cellular compartments including mitochondria^15^,^19^. The liver is considered SAMe's factory, as it is where half of our daily intake of methionine is converted into SAMe and having normal SAMe levels is essential for liver health^15^. Lower SAMe levels are found frequently in chronic liver diseases including ALD and SAMe administration has shown protective effects in multiple ALD murine models^28^,^29^ and a clinical trial of patients with alcoholic cirrhosis^30^.

We and others have demonstrated that SAMe is important for mitochondrial function^8^,^10^,^19^,^28^,^29^. Schober et al. showed that mitochondrial SAMe deletion has a profound negative impact on mitochondrial metabolism as it leads to defects on mitochondrial translation and the downregulation of both nuclear and mitochondrial encoded subunits of the electron transport chain^19^. Until recently SAMe was believed to be only transported into mitochondria through a specific channel encoded by *SCL25A26*^31^ but we found that MATα1, the catalytic subunit of the enzyme responsible for SAMe biosynthesis in hepatocytes, is also present in mitochondria of hepatocytes and is another source of mitochondrial SAMe^8^,^10^. We have observed that in hepatocytes, mitochondrial MATα1 deficiency leads to lower mitochondrial membrane potential, higher production of mROS and reduced respiration^8^,^10^. Additionally, we have identified dozens of mitochondrial proteins interact with MATα1, likely to facilitate their methylation. We described that MATα1 negatively regulates CYP2E1 expression through methylation^8^. We recently found that ethanol selectively depletes mitochondrial MATα1 in hepatocytes and that its preservation improves mitochondrial function in correlation with higher methylation and expression of mitochondrial proteins^10^. Taken together these data demonstrate that mitochondrial MATα1 is important to maintain mitochondrial proteome and function in hepatocytes. In recent years, an increasing number of mitochondrial proteins have been found to be methylated and multiple mitochondrial protein methyltransferases have been identified. Human mitochondrial DNA (mtDNA) is extensively methylated^32^ and it has been suggested that methylation plays a role in mtDNA replication and gene expression^33^,^34^. All these findings support that the methylation status is crucial for mitochondrial biology.

Since *Mat1a*-KO mice have reduced hepatic SAMe level and mitochondrial dysfunction^8^, we investigated whether SAMe level might regulate MCJ functionality. We found higher MCJ expression in *Mat1a*-KO mice mostly at the protein level even in young mice. There are different mechanisms by which low SAMe level causes mitochondrial dysfunction and one of them is reduced expression of the mitochondrial chaperone PHB1^35^. PHB1 stabilizes newly synthesized mitochondrial proteins and its deficiency is associated with mitochondrial defects^36^. Deletion of *Phb1* in hepatocytes causes mitochondrial abnormalities and liver injury^37^. To examine if PHB1 could be involved in MCJ upregulation in *Mat1a*-KO mice, we assessed MCJ levels in liver-specific *Phb1*-KO mice and found normal hepatic MCJ levels in these mice, excluding PHB1 as a regulatory mechanism of MCJ expression.

Higher MCJ protein levels were confirmed in other models of reduced SAMe levels such as *Mat1a* silenced and dedifferentiating hepatocytes. This regulation is clinically relevant, as patients with chronic liver diseases including ALD and NASH have lower hepatic expression of MAT1A and SAMe levels^15^. On the other hand, we observed that SAMe treatment and MATα1 overexpression reduced MCJ protein levels in different liver cells but the MATα1 catalytic mutant did not, which suggests that MATα1 regulates MCJ via SAMe. Supporting this, we also observed SAMe exerted a strong downregulation of MCJ expression in cells lacking MAT1A such as *Mat1a*-KO hepatocytes and SAMe-D cells. Given the fact that MCJ is ubiquitously expressed^1^ and SAMe is present in all mammalian cells^15^, we evaluated whether this regulation also occurs outside hepatocytes. We found that SAMe treatment reduced MCJ protein levels in hepatic stellate cells, macrophages and colon and breast cancer cells. While this finding is intriguing, we believe that this mechanism is particularly relevant in the liver because the liver is one of the organs where MCJ is expressed at a higher level^1^; MCJ is upregulated in multiple liver diseases including drug-induced liver injury^2^, non-alcoholic fatty liver disease^3^, cholestatic liver disease^4^, and alcohol-associated liver disease; the liver serves as the body's primary SAMe factory^15^, and SAMe levels are diminished in liver diseases^15^, including those mentioned above; and lastly, because the administration of SAMe has shown protective effects in these conditions^15^.

Because MATα1 promotes CYP2E1 methylation within mitochondria and proteasomal degradation of methylated CYP2E1^8^, we studied whether MCJ was regulated the same way. We found that MATα1 interacts with MCJ in liver mitochondria and this may promote MCJ methylation. Several methylation prediction softwares identified five highly scored potential lysine methylation sites in the MCJ protein sequence and we found that MCJ is methylated at lysine residues. MCJ co-IP followed by MS allowed us to identify lysine 83 (85 in human MCJ) di-methylation which was markedly reduced in *Mat1a*-KO mouse liver. The upregulation and hypomethylation of MCJ protein in *Mat1a*-KO liver strongly suggest that methylation regulates its expression in the liver. This led us to further investigate whether MCJ stability is regulated by methylation at key lysine residues. We mutated lysine 83 and the other predicted lysines to alanines to prevent their methylation and evaluated the effect of SAMe on MCJ protein levels. We did not see any difference as compared to WT MCJ, suggesting that the mechanism by which SAMe regulates MCJ might involve more than just methylation at a single lysine residue. It is possible that 1) multiple methylation sites are involved in MCJ, 2) other post-translational modifications (PTMs) are involved such as phosphorylation and sumoylation, both of which are inhibited by SAMe^20^,^21^,^38^, 3) an indirect effect on another protein to enhance that protein's ability to degrade MCJ. Although the outcome has not been studied, multiple phosphorylation and glycosylation sites in MCJ have been identified in high-throughput studies and we found several SUMO interaction motifs predicted in its sequence.

PTMs, including methylation, occur in various cellular compartments. It has been suggested that mitochondrial proteins that are synthesized in the cytoplasm can undergo methylation before they are transported to mitochondria or can be modified within the organelle^39^. Certain methyltransferases are present in the mitochondrial matrix or associated with the inner membrane of the mitochondria where they can methylate specific proteins^39^. MCJ is a nuclear-encoded protein, and it is possible that it is methylated before being transported into the mitochondria. However, due to its short half-life, which is further shortened by SAMe, we believe that the process occurs inside mitochondria, but this remains to be confirmed. In recent years, an increasing number of mitochondrial proteins have been found to be methylated and multiple mitochondrial protein methyltransferases have been identified^39^. If methylated within mitochondria, some potential lysine methyltransferases include METTL12, METTL20, METTL21D, FAM173A, and FAM173B^39^. These lysine methyltransferases play crucial roles in modulating the function, stability, and interactions of mitochondrial proteins although their specific targets are still being elucidated.

Although we did not identify how SAMe regulates MCJ, we confirmed that its effect relies on destabilizing MCJ, as it significantly reduced the protein's half-life. Interestingly, SAMe induced a rapid depletion of MCJ in all cells during the first 30 minutes of the cycloheximide chase experiment and after this time, the fall seemed slower than control, suggesting that there is a pool of MCJ that is particularly sensitive from SAMe depletion. These findings along with unchanged MCJ translation in Huh-7 treated with SAMe further support that SAMe regulates MCJ post-translationally.

Mitochondrial proteome homeostasis is complex. Mitophagy, the ubiquitin-proteasome system, and the mitochondrial chaperone and protease machinery are the three major mechanisms regulating mitochondrial protein degradation^22^. As there are no good approaches to inhibit mitochondrial chaperones or proteases unless targeting each enzyme individually, we explored the lysosomal and proteasomal degradation pathways. Mitophagy followed by lysosomal degradation and mitochondrial export followed by proteasomal degradation are known mechanisms by which mitochondrial proteins are degraded^23^. We found that MCJ is not degraded by either of these mechanisms as its protein level was further reduced instead of accumulated when either the proteasome or autophagy was blocked. MG132 treatment raised MATα1 level, and we hypothesize that might be the explanation for the fall in MCJ, but we found the same result in *Mat1a*-KO hepatocytes, suggesting that the effect of MG132 is independent of MATα1. CQ, the autophagy inhibitor, did not raise MATα1 protein, ruling out the involvement of MATα1 in its effect. SAMe addition further reduced MCJ expression in both MG132 and CQ treated mouse hepatocytes. An explanation for the further reduction in MCJ is that a key protein (or proteins) that regulate(s) MCJ stability accumulated when the proteasome or autophagy was blocked and promoted the rapid degradation of MCJ.

Mitochondria possess a complex network of proteases that together with chaperones remove proteins that have become nonfunctional or damaged to relieve stress and re-establish homeostasis^22^. Based on our results, the most plausible explanation is that MCJ degradation involves the mitochondrial protease machinery and SAMe likely enhances it via methylation and/or other PTMs. Compared to mitophagy, this pathway allows a selective recognition and degradation of specific proteins^22^. Additionally, protein degradation by mitochondrial proteases that work directly within the mitochondria allows for a relatively quick response to clear malfunctioning proteins, which could be consistent with MCJ's short half-life. Because of the complexity of this pathway -there are around 45 known mitochondrial proteases-, determining how MCJ is degraded is beyond the scope of the study and remains a goal for future analysis.

In this work, we show that MCJ upregulation is another mechanism that contributes to mitochondrial dysfunction in conditions of MAT1A and SAMe deficiency. While SAMe and MAT1A deficiency can lead to changes in mitochondrial content that could alter the expression of mitochondrial proteins, we did not find a significant alteration in the overall content of mitochondria in our experiments, suggesting that the effect of SAMe on MCJ is specific and not due to global mitochondrial changes. We observed that the negative impact of *MAT1A* silencing on mitochondrial function in HepG2 cells can be partially reverted by *DNAJC15* silencing as seen by higher ATP levels and mitochondrial respiration. The fact that mitochondrial function was not fully recovered suggests that other mechanisms are involved in MAT1A and SAMe deficiency-induced mitochondrial dysfunction but overall, our results demonstrate that MCJ plays a major role. We also evaluated the impact of MCJ on the mitochondrial function of *Mat1a*-KO hepatocytes. We observed that *Dnajc15* silencing improved it to the levels of WT, supporting the fact the MCJ upregulation is a major mechanism of mitochondrial dysfunction in these mice. The production of ATP, basal respiration and maximal respiratory capacity were all improved to the levels of WT.

*DNAJC15* silencing had no impact on mROS production at baseline and did not reduce the increase in mROS caused by *MAT1A* silencing in HepG2 cells, or in WT and *Mat1a*-KO hepatocytes. The relationship between MCJ with ROS seems complicated as opposite observations have been made in different cell types and conditions. MCJ deficiency has been linked to enhanced formation of mitochondrial supercomplexes that attenuate the leak of electrons and production of mROS in CD8 T cells^1^,^40^ and hepatocytes^2^ but macrophages from *Mcj*-KO mice have higher mitochondrial function and mROS^41^. In hepatocytes, MCJ deficiency seems to not affect mROS production at baseline but reduces its formation upon hepatotoxic stimuli such as ethanol.

ALD represents a wide spectrum of diseases, ranging from the relatively benign hepatic steatosis to cirrhosis and HCC, with AH being the most aggressive form of ALD^24^. A surge in the use of alcohol and incidence of ALD has been observed in the last few years but there are no FDA-approved therapies for its treatment and current treatments involve mainly lifestyle changes^24^. Since alcohol-induced mitochondrial injury is an important event in the onset of ALD^9^ and mitochondrial MATα1 is selectively depleted in ALD^10^, we investigated whether MCJ might have a role in the pathogenesis of the disease. We found that MCJ is upregulated in human ALD, and preclinical models of ALD such as livers from ethanol-fed mice following the NIAAA mouse model and AML-12 cells treated with ethanol. The NIAAA model consists of a chronic-plus-single-binge feeding and induces liver injury, inflammation and fatty liver, mimicking acute-on-chronic alcoholic liver injury in patients^25^. The importance of MCJ in ALD was demonstrated by knocking down MCJ in hepatocytes prior to treatment with ethanol. *Dnajc15* silencing raised the production of ATP, reduced ethanol-induced production of mROS, improved mitochondrial respiration and ultimately, reduced the accumulation of triglycerides in hepatocytes. Furthermore, we observed that SAMe administration, which is known to be beneficial in ALD, was able to inhibit ethanol-induced MCJ upregulation in AML-12 cells. Altogether, these results conclude that MCJ is an important mediator of mitochondrial dysfunction in ALD and a promising target for its treatment.

In summary, our study shows that SAMe is an inhibitor of MCJ in the liver by reducing MCJ protein stability. MCJ upregulation represents another mechanism by which SAMe deficiency promotes liver injury and a new targetable mechanism in alcohol-induced mitochondrial injury. This work illustrates the importance of MATα1 and SAMe in mitochondrial metabolism.

## Materials and Methods

### Human Samples

Slides containing liver specimens from subjects with severe alcoholic hepatitis (AH) (n=3) were obtained from Johns Hopkins University from the explant during liver transplantation. Liver samples from controls (n=3) were from subjects with no known history of excessive alcohol use or underlying chronic liver disease. They were obtained during abdominal surgeries for various causes. All liver samples were obtained under a protocol approved by the Human Subjects and Institutional Review Board at Johns Hopkins University. Written informed consent was obtained from each participant.

### Mice

Two and a half- and four-month-old male *Mat1a*-KO (C57Bl/6)^13^ and liver-specific *Phb1*-KO (C57Bl/6)^37^ and age-matched wild-type (WT) male sibling littermates were used for this study. Animals were bred, maintained, and cared for as per National Institutes of Health (NIH) guidelines, and protocols were approved by the Institutional Animal Care and Use Committee of Cedars-Sinai Medical Center, Los Angeles, CA. The animals were euthanized using isoflurane anesthesia in a desiccator jar followed by exsanguination and organ harvest under sterile conditions. Mice (n=5/group) were treated with ethanol or pair-fed control diet following the protocol of the National Institute on Alcohol Abuse and Alcoholism (NIAAA) model^25^. Briefly, mice were fed ad libitum with the Lieber-DeCarli liquid diet (Bio-Serv) for 5 days and then divided into two groups: the ethanol group was fed a liquid diet containing 5% ethanol for 10 days and the control group was pair-fed control diet for 10 days. At day 11, mice in the ethanol group received a single binge ethanol feeding (5g/kg, 20% ethanol) while mice in the control group were received dextrin maltose. The gavage was performed early in the morning and after gavage, mice were kept on control or ethanol diet and in the cages with water. The mice were euthanized 9 hours after the gavage. All procedure protocols, use, and the care of the animals were reviewed and approved by the Institutional Animal Care and Use Committee at Cedars-Sinai Medical Center. All mice are housed under 12-hour light/12-hour dark cycle at an average temperature of 74F and 40% humidity. For MCJ characterization studies we used n=5 WT and *Mat1a*-KO mice and n=7 WT and *Phb1*-KO mice.

### Cell culture and treatments

Human HCC cell lines HepG2, Huh-7, RKO, and HT-29, and the mouse hepatocyte cell lines AML-12 and RAW 264.7 were purchased from American Type Cell Collection (ATCC, Manassas, VA. LX-2 cells were kindly provided by Dr. Ekihiro Seki and MCF-7 cells were kindly provided by Dr. Mercedes Rincon. The SAMe-D cell line, derived from HCC of a *Mat1a*-KO mouse, was previously described^16^. HCC cell lines were cultured in DMEM containing 10% fetal bovine serum (FBS) and antibiotics (2 mM glutamine, 50 mM penicillin, and 50 mg/ml streptomycin sulfate) and AML-12 cells were grown in DMEM-F12 containing a cocktail of insulin, selenium, and transferrin (41400-045 Thermo Scientific), 0.05 μg/mL dexamethasone (D4902 Sigma), 10% FBS and antibiotics. All cell lines were authenticated, using short tandem repeat profiling (ATCC; Manassas, VA).

Primary mouse hepatocytes were isolated following the standard two-step collagenase perfusion technique. Briefly, mice received a terminal dose of anesthetic and underwent laparotomy^42^. The portal vein was cannulated and then perfused with 50 mL Liver Perfusion Medium (Gibco) followed by 50 mL Liver Digest Medium (Gibco) pre-warmed to 37 °C. The liver was removed and then hepatocytes were mechanically disassociated and released from the Glisson capsule, filtered through 70 μM filter and purified with a density gradient centrifugation. The cells were seeded at a concentration of 5×10^4^ cells/6-well plate on collagen- or matrigel-coated dishes. Cryopreserved human hepatocytes were obtained from different donors (Thermo Scientific) and stocked in liquid nitrogen. The vials were thawed in a water bath and cells were plated on matrigel-coated dishes. After 3 hours, the medium was changed and cultures were maintained in MEM containing 10% FBS and antibiotics. Cells cultured in 6-well plates (0.2 million per well) were treated with different doses of SAMe for different times. Cells were treated with MG132 20 μM and chloroquine (CQ) 50 μM 6 hours before harvesting. AML-12 cells were treated with ethanol (E7023 Sigma) 100 mM (twice a day) for 48 hours. For SAMe co-treatment, AML-12 cells were treated with SAMe 500 μM for 48 hours (once a day). For cycloheximide (CHX) chase experiments, cells were treated with SAMe for 24 hours. The media was changed to serum free prior to the addition of 10 μg/mL CHX for 0, 15 min, 30 min, 1, 2, and 3 hours.

### Gene overexpression

For gene overexpression experiments, 1x10^5^ (6 well-plate) Huh-7 or SAMe-D cells were transiently transfected with MAT1A WT (GeneCopoeia), MAT1A catalytic mutant, HA-tagged MCJ WT (provided by Dr. Mercedes Rincon), HA-tagged MCJ K72A, K83A, K89A, K107A and K125A overexpression vectors or empty vector using JetPrime® (Polyplus) according to the manufacturer's protocol. 1.6 µg of target plasmid per 6-well plate were used for transfection overnight. Next morning medium was changed to normal medium. The cells were cultured for additional 48 hours for protein expression mRNA and analysis.

### Gene silencing

For *MAT1A* knockdown 50 nM siRNA against human *MAT1A* (SR303529, Origene) or mouse *Mat1a* (161996, Thermo Scientific) or equivalent scramble control (SC) were delivered into mouse hepatocytes or HepG2 cells by Lipofectamine RNAiMAX (Life Technologies) for 48 hours following the manufacturer's protocol. For *DNAJC15* knockdown, 20 nM siRNA against human *DNAJC15* (133982, Thermo Scientific), or mouse *Dnajc15* (s82673, Thermo Scientific), or equivalent scramble control (SC) were delivered into HepG2 or AML-12 cells or primary hepatocytes following the same protocol describe above. Primary hepatocytes were transfected overnight, the media was changed the next morning, and the cells were collected 24 hours later.

### Site directed mutagenesis and DNA sequencing

For the generation of MCJ mutants, primers were purchased from Eurofins Genomics. Primers were as follows: K83A_F: 5' GCTTTTCATCCTACTATGCAGGAGGATTCGAGCAG 3'; K83A_R: 5' CTGCTCGAATCCTCCTGCATAGTAGGATGAAAAGC 3'; K72A_F: 5' CGGCAACAGCAAGGGCGATTTCCTCTCCAAG '; K72A_R: 5' CTTGGAGAGGAAATCGCCCTTGCTGTTGCCG 3'; K89A_F: 5' GGAGGATTCGAGCAGGCAATGAGTAAGCGAG 3'; K89A_R: 5' CTCGCTTACTCATTGCCTGCTCGAATCCTCC 3'; K107A_F: 5' GTAAGCCCATCTGCTGGCGCGGCCAAGATTAGAACAG 3'; K107A_R: 5' CTGTTCTAATCTTGGCCG CGCCAGCAGATGGGCTTAC 3'; K125A_F: 5' GATTTTAAACCATCCAGACGCAGGTGGATCTCCTTACTTAG 3'; K125A_R: 5' CTAAGTAAGGAGATCCACCTGCGTCTGGATGGTTTAAAATC 3'. The quick-change lightning multi-site-directed mutagenesis kit (Agilent Technologies) was used to generate mutants. Mutations were confirmed through sequencing by GENEWIZ.

### Subcellular fractionation

To extract mitochondria from liver the Mitochondria Isolation Kit for Tissue (ab110168) was used following manufacturer's instructions. Briefly, 50-100 mg of livers were wash twice with 1.5 mL of Wash Buffer. Tissue was minced, placed in pre-chilled Dounce homogenizer and homogenized (30 strokes) in 1mL of isolation buffer. Homogenates were taken to 2 mL with isolation buffer and centrifuged at 1,000 x *g* for 10 minutes at 4°C. Supernatants were centrifuged at 12,000 x *g* for 15 minutes at 4°C. Supernatant (cytosolic fraction) was collected and pellets (mitochondrial fraction) were washed with 1 mL of isolation buffer and centrifuged one more time at 12,000 x *g* for 15 minutes at 4°C. Pellets were collected and resuspended in isolation buffer supplemented with protease inhibitor cocktail.

### Cell lysis and western blotting

For western blotting, 10-30 μg of whole cell extract, cytoplasmic protein or mitochondrial extract were separated by 10% SDS-PAGE. Blots were probed with antibodies diluted 1:1000 in 5% skim milk against MATα1 (ab129176), MCJ (antibodies provided by Dr. Mercedes Rincon), HA-tag (3724 Cell Signaling), COXIV (4844 Cell Signaling), OXPHOS (ab110413), GAPDH (5174 Cell signaling Technology) and β-actin (A3854 Sigma). HRP-linked secondary antibodies anti-rabbit (7074S Cell Signaling) and anti-mouse (7076S Cell Signaling) were diluted 1:5000 in 5% skim milk. Images were quantified by densitometry using the ImageJ densitometry program (National Institutes of Health, Bethesda, MD).

### RNA isolation and RT-PCR

Total RNA was isolated with Trizol (Invitrogen). Total RNA (1-2 µg) was reverse transcribed into cDNA using M-MLV Reverse Transcriptase (Invitrogen). Two microliters of RT product were subjected to quantitative real-time PCR analysis. TaqMan probes for human *MAT1A* (Hs01547962_m1), DNAJC15 (Hs00387763_m1)*,* and murine *Mat1a* (Mm00522563_m1), *Dnajc15* (Mm00481271_m1) and *Phb1* (Mm01627033) were purchased from Applied Biosystems. Universal PCR Master Mix was purchased from Bio-Rad. Ubiquitin C was used as housekeeping gene (Hs01871556_s1 and Mm02525934_g1). The cycle Ct value of the target genes was normalized to that of the housekeeping gene to obtain the delta Ct (∆Ct). The ΔCt obtained was used to find the relative expression of target genes according to the formula: relative expression = 2-ΔΔCt, where ΔΔCt = ΔCt of target genes in experimental groups - ΔCt of target genes in control group.

### Polysome profiling

Huh-7 cells were treated with SAMe and total RNA was fractionated for polysome profiling as described^21^ and real-time PCR for *DNAJC15*. Data represent the mRNA distribution and polysome/ NTR ratio.

### Immunohistochemistry

Paraffin-embedded sections of human liver tissue were subjected to antigen retrieval using a citric acid-based heating method (Abcam, Cambridge, MA). Slides were incubated with MCJ antibody (1:100, overnight at 4 °C) followed by 1 hour with anti-mouse reagents and developed using the HRP/DAB detection immunohistochemistry kit (Abcam). Stained slides were imaged at 20X and 40X magnification using an inverted microscope (EVOS XL core, Life technologies).

### Immunoprecipitation

700 μg of whole liver or cells extracts or 350 μg of mitochondrial fractions were pre-cleaned by adding 0.7 μg of the appropriate normal Ig together with 20 μl of appropriate protein A+G-agarose conjugate (Santa Cruz Biotechnology, Dallas, TX) for one hour at 4°C. The IPs were performed with 3 μg of anti-MATα1, anti-MCJ, anti-methyl-lysine (ab23366) or anti-methyl-arginine (ab412) antibody overnight at 4°C. For the MS studies, a total of 1,000 μg whole cell lysate was used to co-IP with antibody against MCJ. In all cases, the protein complexes bound to the protein A+G-agarose conjugate were washed five times with IP buffer (150 mM NaCl, 0.5% deoxycholate, 0.1% SDS, 1% NP-40, 50 mM Tris-HCl, pH 7.5) with protease inhibitors and then diluted in IP buffer (50 mM Tris-HCl, 150 mM NaCl, 2 mM EDTA, 2 mM EGTA, 25 mM NaFl, 25 mM β- glycerophosphate (pH 7.5), 0.1 mM sodium orthovanadate, 0.1 mM PMSF, 5 μg of leupeptin per ml, 0.2% (vol/vol) Triton X-100, 0.5% (vol/vol) Nonidet P-40). Sample was process for MS as outlined below or separated on by 10% SDS-PAGE for immunoblot. The gel was transferred to nitrocellulose membrane and probed with antibodies as described above. TrueBlot IP Detector Reagent (Rockland) was used to reduce background. Normal Ig (Cell Signaling) was used as a control.

### Direct protein-protein interaction

Recombinant human MATα1 and MCJ proteins were from ProSpec. 2 μg of MATα1 or MCJ recombinant protein was immobilized to agarose beads by their respective antibody (MAT1A Monoclonal antibody Cat no: 67408-1-Ig). After washing, beads were mixed with 1 μg MATα1 or MCJ protein and rotated for four hours at 4°C. Beads were then washed six times in binding buffer (50 mM Tris-HCl pH 7.5, 0.2 mm EDTA, 0.25 mM PMSF, 0.5% NP-40), boiled in SDS sample buffer and proteins separated on 10% SDS-PAGE and subjected to western blotting. IgG was used as negative control.

### Mitochondrial ROS

Mitochondrial ROS was measured using the MitoSOX Red kit (M36008 Molecular Probes) according to the manufacture's guide. In brief, cells were incubated with MitoSOX 5 µM in HBSS medium without FBS for 15 minutes in a CO_2_ incubator at 37ºC. Then cells were washed twice with warmed PBS, fluorescence was measured at Ex/Em: 510/580 nm and normalized to total protein. Mitochondrial ROS was also evaluated by fluorescence microscopy.

### ATP levels

The levels of ATP were determined using the ATPlite luminescence ATP detection assay system (PerkinElmer) according to the manufacture's guide. Measurement was normalized to total protein.

### Mitochondrial respiration

Intact cellular respirometry was conducted using Seahorse XF^e^96 and XF^e^24 extracellular flux analyzers. HepG2 and AML-12 cells and were seeded (10,000 cells/well) onto 96-well XF cell culture plates and transfected using the protocol described above. Primary mouse hepatocytes were seeded (20,000 cells/well) onto 24-well XF cell collagen-coated culture plates and transfected using the protocol described above. After transfection, HepG2 were cultured for 48 hours, AML-12 cells were treated with ethanol for 48 hours as described above, and primary hepatocytes we cultured for another 24 hours. The day of the assay the cells were refreshed with bicarbonate-free DMEM containing 5.5mM glucose, 1mM sodium pyruvate, 4mM glutamine and equilibrated for 1h at 37°C in a non-CO_2_ incubator. Oxygen consumption was subsequently monitored following sequential injection of oligomycin (4µM), FCCP (1µM) & antimycin/rotenone (2µM/2µM). For normalizing respiration rates, cells were subsequently lysed in lysis buffer and protein concentration was determined using the BCA assay. The experiments were performed 3 times.

### Triglyceride levels measurement

Triglycerides were measured using the Triglyceride Colorimetric Assay Kit from Cayman (No. 10010303) according to the manufacture's guide. Measurement was normalized to total protein.

### Cell viability

To assess cell viability, cell suspension was mixed with equal volumes of trypan blue dye and cells were counted using a hemocytometer. The total cell number and the viable cells that excluded trypan blue were counted. The viability was represented as the percentage of trypan blue-excluded cells versus total cells.

### Mass spectrometry acquisition methods

(DDA-MS) Data-dependent acquisitions and data-independent acquisition mass spectrometry (DIA-MS) acquisitions were performed on an Orbitrap LUMOS Fusion mass spectrometer equipped with an EasySpray ion source and connected to an Ultimate 3000 nano LC system with a 60-min gradient. Peptides were loaded onto a PepMap RSLC C18 column (2 µm, 100 Å, 150 µm i.d. × 15 cm, Thermo) using a flow rate of 1.4 µL/min for 7 min at 1%B (mobile phase A was 0.1% formic acid in water and mobile phase B was 0.1% formic acid in acetonitrile) after which point, they were separated with a linear gradient of 5-20%B for 45 min, 20-35%B for 15 min, 35-85%B for 3 min, holding at 85%B for 5 min, and re-equilibrating at 1%B for 5 min. Each sample was followed by a blank injection to both clean the column and re-equilibrate at 1%B. The nano-source capillary temperature was set to 300 °C and the spray voltage was set to 1.8 kV. For DIA analysis, MS1 scans were acquired in the Orbitrap at a resolution of 60,000 Hz from mass range 400-1000 m/z. For MS1 scans the AGC target was set to 3 × 10^5^ ions with a max fill time of 50 ms. DIA MS2 scans were acquired in the Orbitrap at a resolution of 15,000 Hz with fragmentation in the HCD cell at a normalized CE of 30. The MS2 AGC was set to 5e4 target ions and a max fill time of 22 ms. DIA was performed using 4 Da (150 scan events) windows over the precursor mass range of 400-1000 m/z and the MS2 mass range was set from 100 to 1500 m/z. For DDA analysis MS1 scans the AGC target was set to 4 × 10^5^ ions with a max fill time of 50 ms. MS2 spectra were acquired using the TopSpeed method with a total cycle time of 3 s and an AGC target of 5 × 10^4^ and a max fill time of 22 ms, and an isolation width of 1.6 Da in the quadrapole. Precursor ions were fragmented using HCD with a normalized collision energy of 30% and analyzed in the Orbitrap at 15,000 K resolution. Monoisotopic precursor selection was enabled and only MS1 signals exceeding 50,000 counts triggered the MS2 scans, with +1 and unassigned charge states not being selected for MS2 analysis. Dynamic exclusion was enabled with a repeat count of 1 and exclusion duration of 15 s.

### MS analysis

For MCJ methylation analysis, first, spectra were obtained from DDA-MS acquisitions of a MCJ immunoprecipitation. Then, Raw DIA-MS data was imported into Skyline (Skyline Daily, version 19.1.1.309), which was then used to visualize and manually validate the co-eluting precursor and fragment trace of a peptide containing Lys83 methylation. Data has been uploaded to Panorama (https://panoramaweb.org/d4I5O5.url).

### Single-cell RNA sequencing

The processed data which was deposited in public data base, NCBI Gene Expression Omnibus (GSE136103) and single cell portal (SCP2153), were used^43^. This processed matrix was loaded into the R with the Seurat package v4.4.0 with meta data including disease ontology and cell type ontology label. Principal component analysis was performed by RunPCA, and dimensionality reduction is conducted by RunTSNE function. The first 15 principal components were used for clustering, and the clusters were marked by cell_type_ontology_label. FeaturePlot and RidgePlot functon were used to visualize the gene expression.

### Statistical analysis

Data are given as mean ± SEM. Statistical analysis was performed using t-tests, ANOVA and Fisher's tests. All P values were derived from at least three independent experiments. Statistical significance was defined by P<0.05. For mRNA and protein levels, the ratios of genes and proteins to respective housekeeping densitometric values were compared. Calculations were performed using Graphpad (version 9.0.0) and Excel. Densitometry analysis were carried out using ImageJ Software (version 1.52q).

## Supplementary Material

Supplementary figures.

## Abbreviations

ALD: alcohol-associated liver disease
AH: alcoholic hepatitis
Co-IP: co-Immunoprecipitation
CQ: chloroquine
CYP2E1: Cytochrome P450 2E1
DILI: drug-induced liver injury
HCC: hepatocellular carcinoma
HSP40: heat shock protein 40
KO: knockout
MASLD: metabolic dysfunction associated steatotic liver disease
MAT1A: methionine adenosyltransferase 1a
MATα1: methionine adenosyltransferase alpha 1
MCJ: Methylation-Controlled J protein
mROS: mitochondrial reactive oxygen species
MS: mass spectrometry
mtDNA: mitochondrial DNA
NASH: non-alcoholic steatohepatitis
NIAAA: National Institute on Alcohol Abuse and Alcoholism
OXPHOS: oxidative phosphorylation
PHB1: prohibitin 1
PTM: post-translational modifications
ROS: reactive oxygen species
SAMe: S-adenosylmethionine
SUMO: Small Ubiquitin-like Modifier
WT: wild type
