# Supplementary material for: S-Adenosylmethionine Negatively Regulates the Mitochondrial Respiratory Chain Repressor MCJ in the Liver
Source: Int J Biol Sci. 2024 Jan 27;20(4):1218–37. doi: 10.7150/ijbs.90104 (PMC10878152; doi:10.7150/ijbs.90104)
Supplement: Supplementary file 1 — Supplementary figures. [file ijbsv20p1218s1.pdf]

SUPPLEMENTARY FIGURE LEGENDS

Supplementary Figure 1. MCJ levels in livers of younger *Mat1a*-KO mice and MAT1A and DNAJC15 expression in human hepatic cells. (A) MATα1 and MCJ protein levels by western blotting and densitometric quantification in liver lysates from 2.5-month-old WT and *Mat1a*-KO mice. (B) *Dnajc15* mRNA levels in livers from 2.5-month-old WT and *Mat1a*-KO mice. (C) Cell identification of human liver cells. t-distributed stochastic neighbor embedding (t-SNE) of single-cell RNA-sequencing (scRNA-seq) 24,548 cells (12,274 cells/group) was generated by Seurat and colored by metadata of disease ontology label in single cell portal. (D) Gene expression of *MAT1A* and *DNAJC15* in t-SNE plot and Ridge plot.

Supplementary Figure 2. MATα1 levels in livers of *Mcj*-KO mice, and MCJ levels in livers of *Phb1*-KO mice. (A) MATα1 levels by western blotting in total and mitochondrial fractions of WT and *Mcj*-KO mouse livers. (B) MCJ levels by western blotting and in liver lysates from WT and liver-specific *Phb1*-KO mice. (C) *Phb1* and (D) *Dnajc15* mRNA levels by RT-PCT in livers from WT and Phb1-KO mice. *P<0.01 *Phb1*-KO vs WT.

Supplementary Figure 3. SAMe lowers MCJ expression in a time- and dose-dependent manner in liver cell lines. MCJ levels by western blotting and quantification (lower panels) in HepG2, Huh-7 and SAMe-D cells treated with (A) SAMe 500 μM for the indicated time points and (B) SAMe at the indicated doses for 24 hours. (C) Viability of HepG2, Huh-7 and SAMe-D cells after SAMe 500 μM treatment for the indicated time points. *P<0.01 SAMe vs control.

Supplementary Figure 4. SAMe reduces MCJ protein levels in other cell types. MCJ protein levels by western blotting and densitometric quantification in RAW, RKO, HT-29, MCF-7, and LX-2 cells treated with SAMe 500 μM for 24 hours. *P<0.01 SAMe vs control.

Supplementary Figure 5. Effect of SAMe on OXPHOS subunits and MCJ translation. (A) Western blot analysis using an OXPHOS antibody cocktail which detects ATP5A (Complex V), UQCRC2 (Complex III), SDHB (Complex II), and NDUFB8 (complex I) in lysates of livers of WT mice, Huh-7, and HepG2 cells treated with SAMe. Densitometric quantification is shown below. (B) *DNAJC15* mRNA translation in polysomes in Huh-7 cells treated with SAMe for 24 hours.

Supplementary Figure 6. MCJ methylation. (A) MCJ lysine and arginine methylation predictions. (B) Spectra of methylated MCJ at Lys83. Representative spectra obtained from DDA-MS acquisitions in MCJ immunoprecipitations showing all of the ions used to identify the peptide containing MCJ Lys83. (C) MCJ-HA levels by western blotting and quantification in Huh-7 cells after MCJ WT, K72A, K83A, K89A, K107A and K125A overexpression for 48 hours and 24 hours of SAMe treatment.

Supplementary Figure 7. MCJ is upregulated in ALD. (A) MCJ levels by immunohistochemistry in all the normal and AH human liver samples at 20x and 40x magnification. (B) *Dnajc15* levels by RT-PCR in livers from pair-fed and ethanol-fed mice following the NIAAA model and (C) AML-12 cells treated with ethanol 100mM for 48 hours. (D) MCJ and MATα1 levels by western blotting in AML-12 cells treated with ethanol 100mM for 24 and 48 hours.

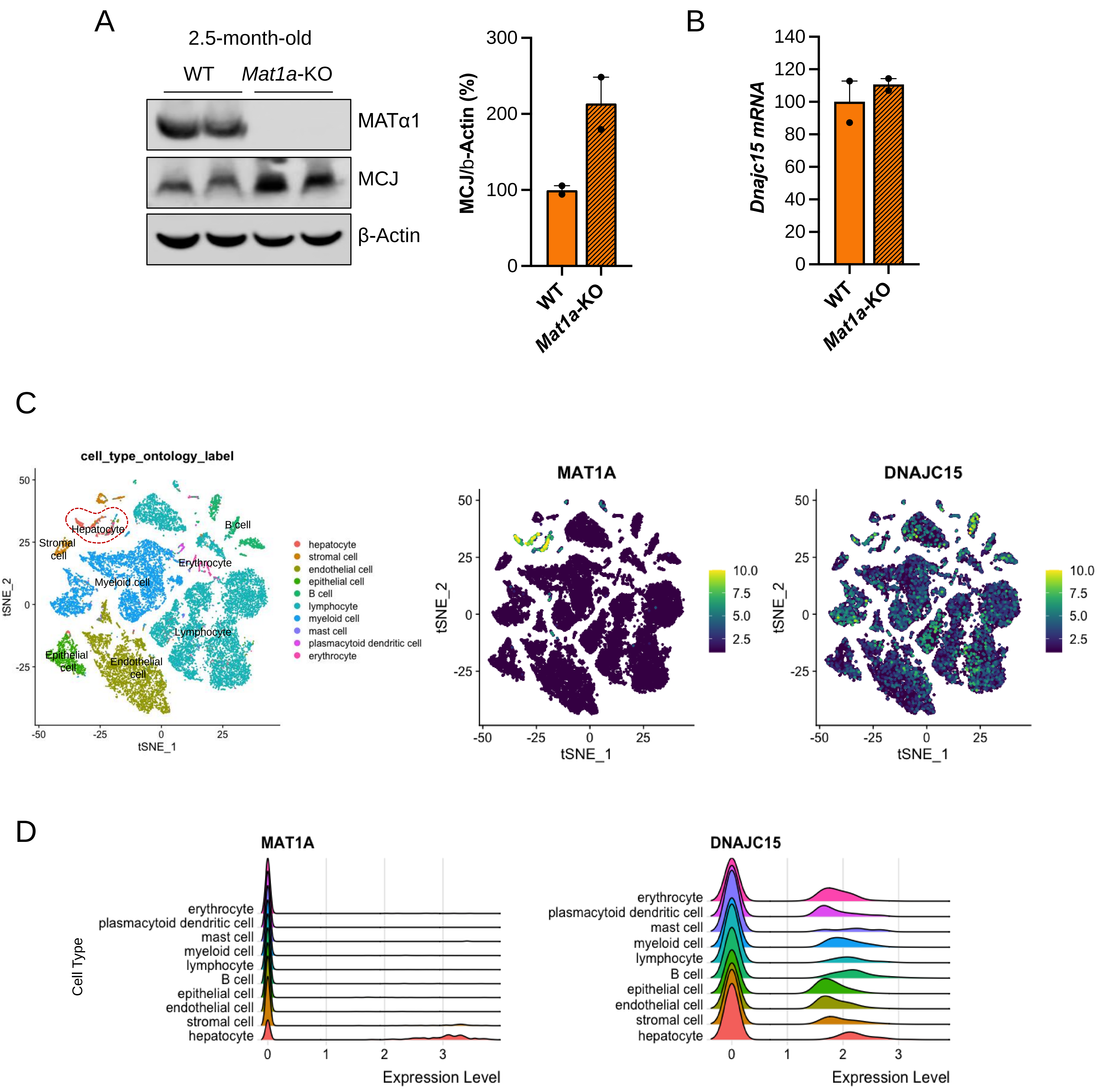
A
2.5-month-old
WT
Mat1a-KO
MATα1
MCJ
β-Actin
MCJ/b-Actin (%)
300
200
100
0
WT
Mat1a-KO
B
Dnajc15 mRNA
140
120
100
80
60
40
20
0
WT
Mat1a-KO
C
cell_type_ontology_label
Hepatocyte
Stromal cell
B cell
Erythrocyte
Myeloid cell
Lymphocyte
Epithelial cell
Endothelial cell
hepatocyte
stromal cell
endothelial cell
epithelial cell
B cell
lymphocyte
myeloid cell
mast cell
plasmacytoid dendritic cell
erythrocyte
tSNE_1
tSNE_2
MAT1A
DNAJC15
10.0
7.5
5.0
2.5
D
MAT1A
DNAJC15
Cell Type
erythrocyte
plasmacytoid dendritic cell
mast cell
myeloid cell
lymphocyte
B cell
epithelial cell
endothelial cell
stromal cell
hepatocyte
Expression Level

Figure S1

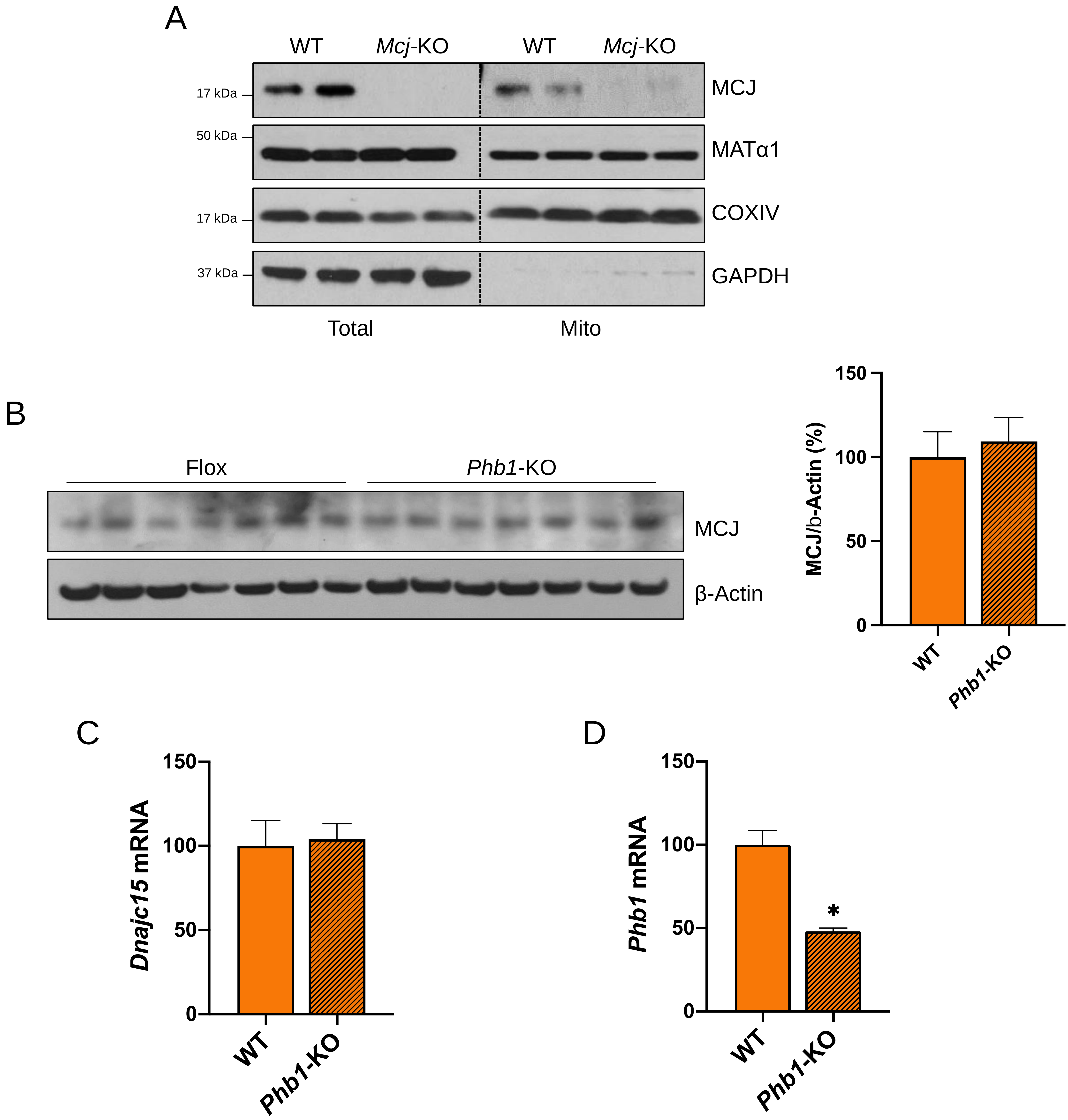
A
WT
Mcj-KO
WT
Mcj-KO
17 kDa
MCJ
50 kDa
MATα1
17 kDa
COXIV
37 kDa
GAPDH
Total
Mito
B
Flox
Phb1-KO
MCJ
β-Actin
MCJ/b-Actin (%)
150
100
50
0
WT
Phb1-KO
C
Dnajc15 mRNA
150
100
50
0
WT
Phb1-KO
D
Phb1 mRNA
150
100
50
0
*
WT
Phb1-KO

Figure S2

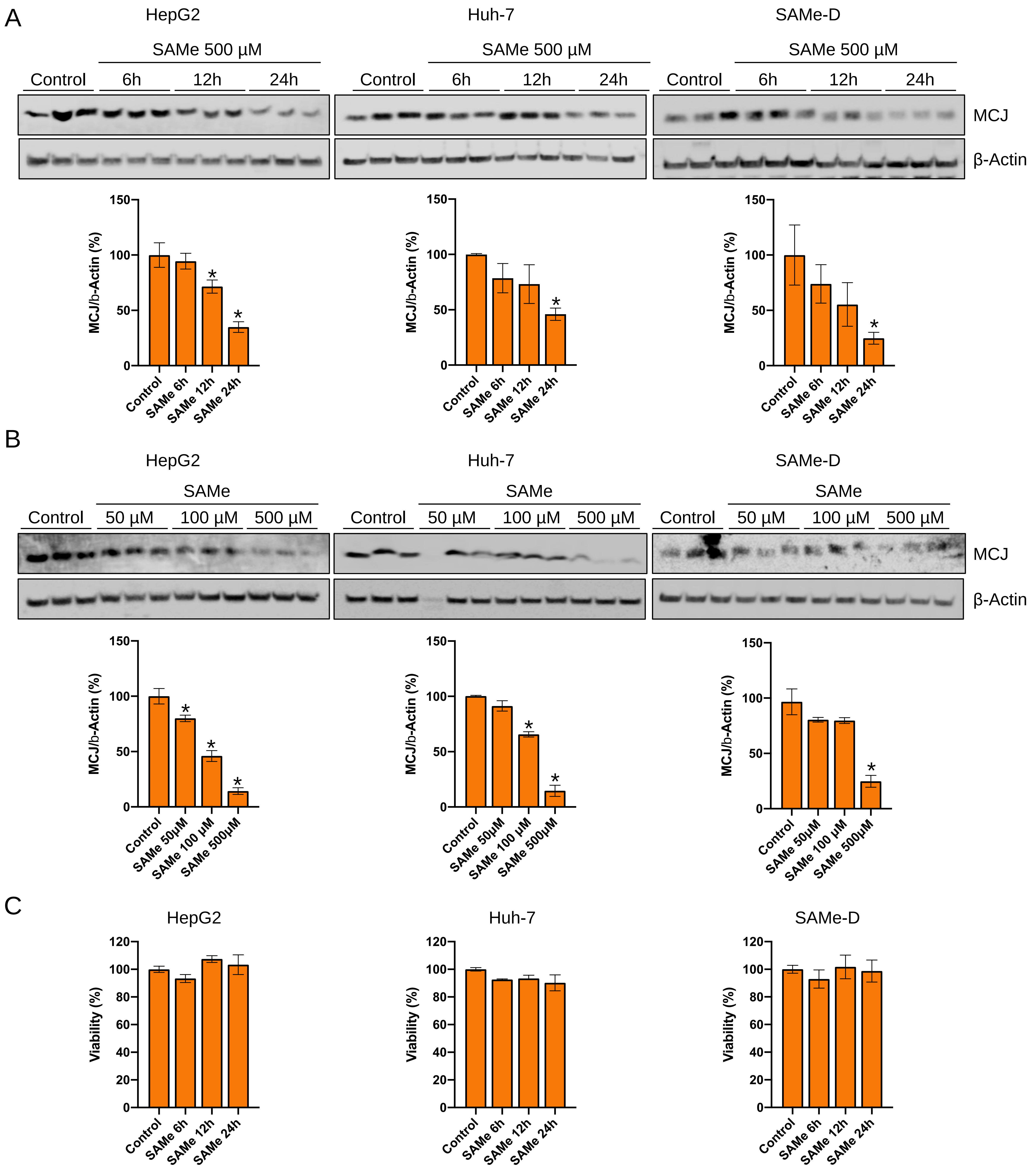
A
HepG2
Huh-7
SAMe-D
SAMe 500 µM
Control
6h
12h
24h
MCJ
β-Actin
MCJ/b-Actin (%)
Control
SAMe 6h
SAMe 12h
SAMe 24h
B
SAMe
50 µM
100 µM
500 µM
SAMe 50µM
SAMe 100 µM
SAMe 500µM
C
Viability (%)

Figure S3

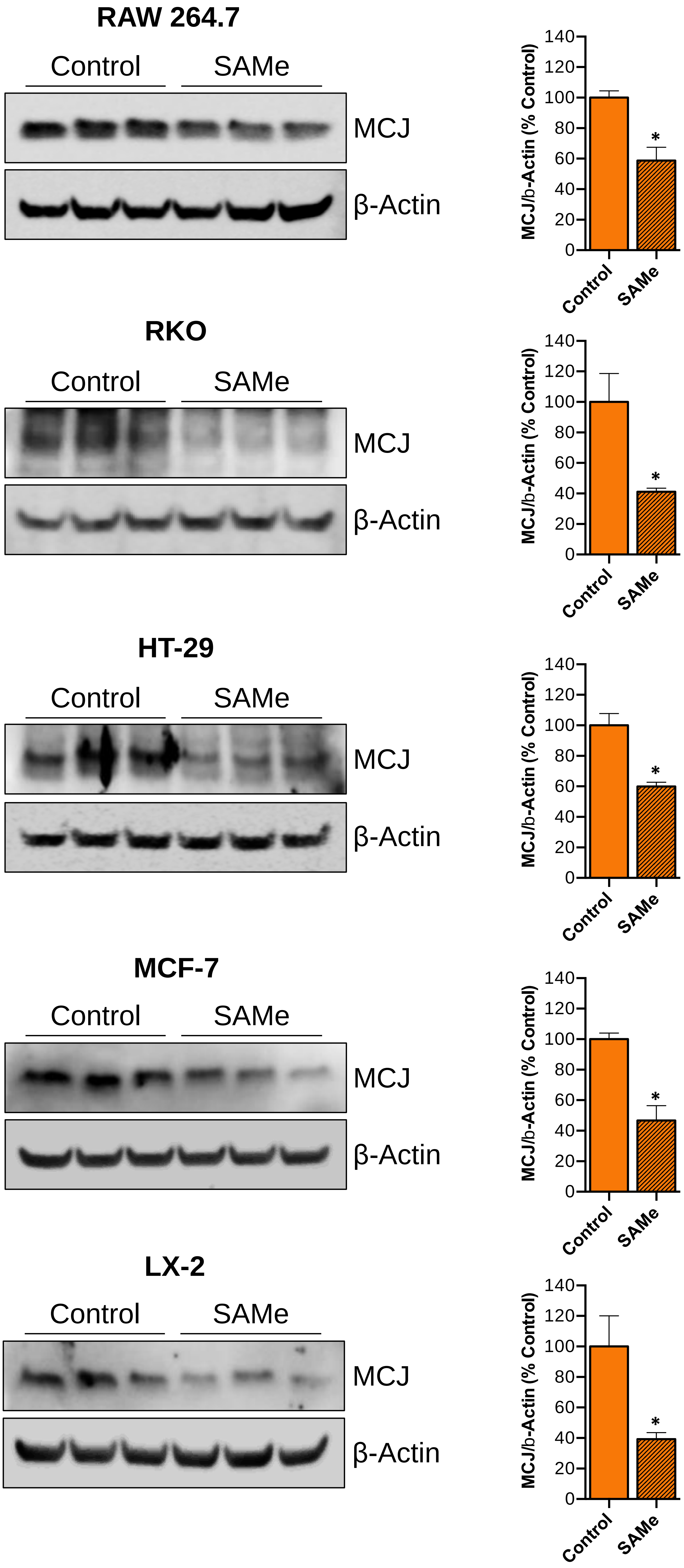
RAW 264.7
RKO
HT-29
MCF-7
LX-2
Control
SAMe
MCJ
β-Actin
MCJ/b-Actin (% Control)
0
20
40
60
80
100
120
140
*

Figure S4

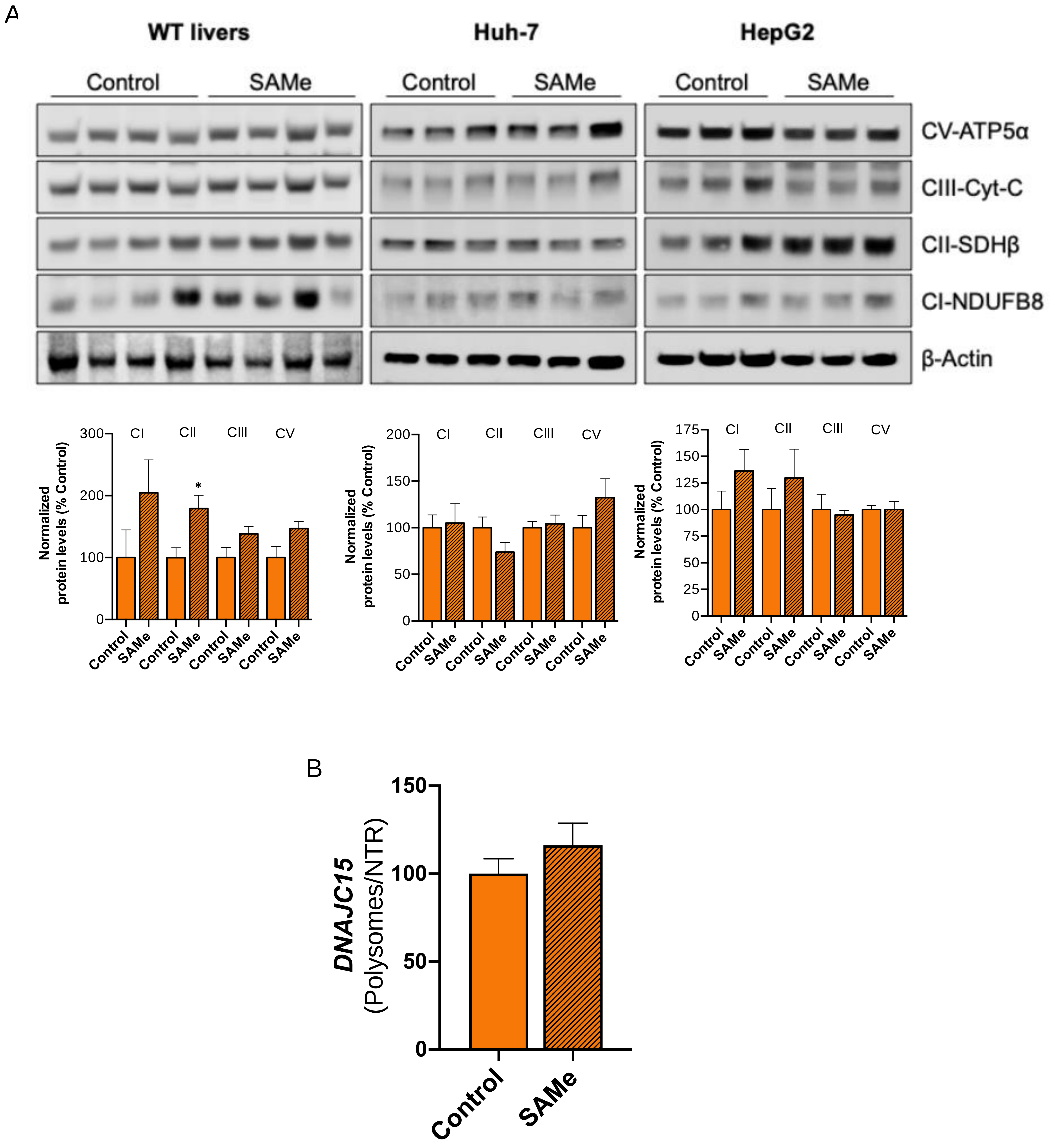
A
WT livers
Huh-7
HepG2
Control
SAMe
CV-ATP5α
CIII-Cyt-C
CII-SDHβ
CI-NDUFB8
β-Actin
Normalized protein levels (% Control)
CI
CII
CIII
CV
*
B
DNAJC15 (Polysomes/NTR)
Control
SAMe

Figure S5

A

Lysine-methylation (Hs/Mm)		
**GPS-MSP**	**Malab**	**PSSME**
25/23	25/23	-
74/72	74/72	74/72
85/83	85/83	85/83
91/89	91/89	91/89
109/107	109/107	109/107
111/109	-	111/109
127/125	127/125	127/125
149/147	-	149/147

Arginine-methylation (Hs/Mm)		
**GPS-MSP**	**Malab**	**PSSME**
4/2	-	4/2
35/33	-	35/33
55/53	-	55/53
94/92	-	94/92

B

Representative Spectra for ISSPSFSSYY**K**GGFEQK

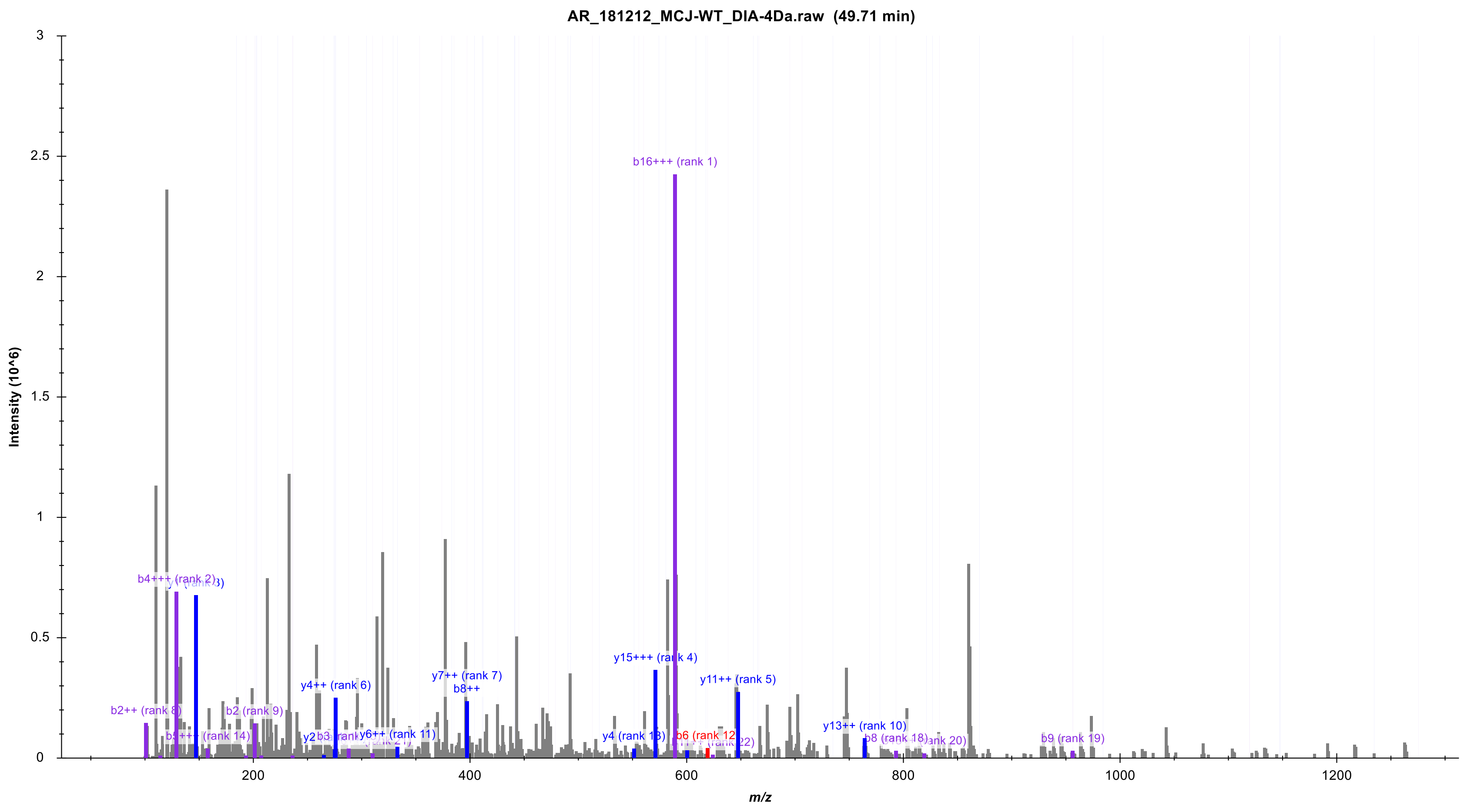

C

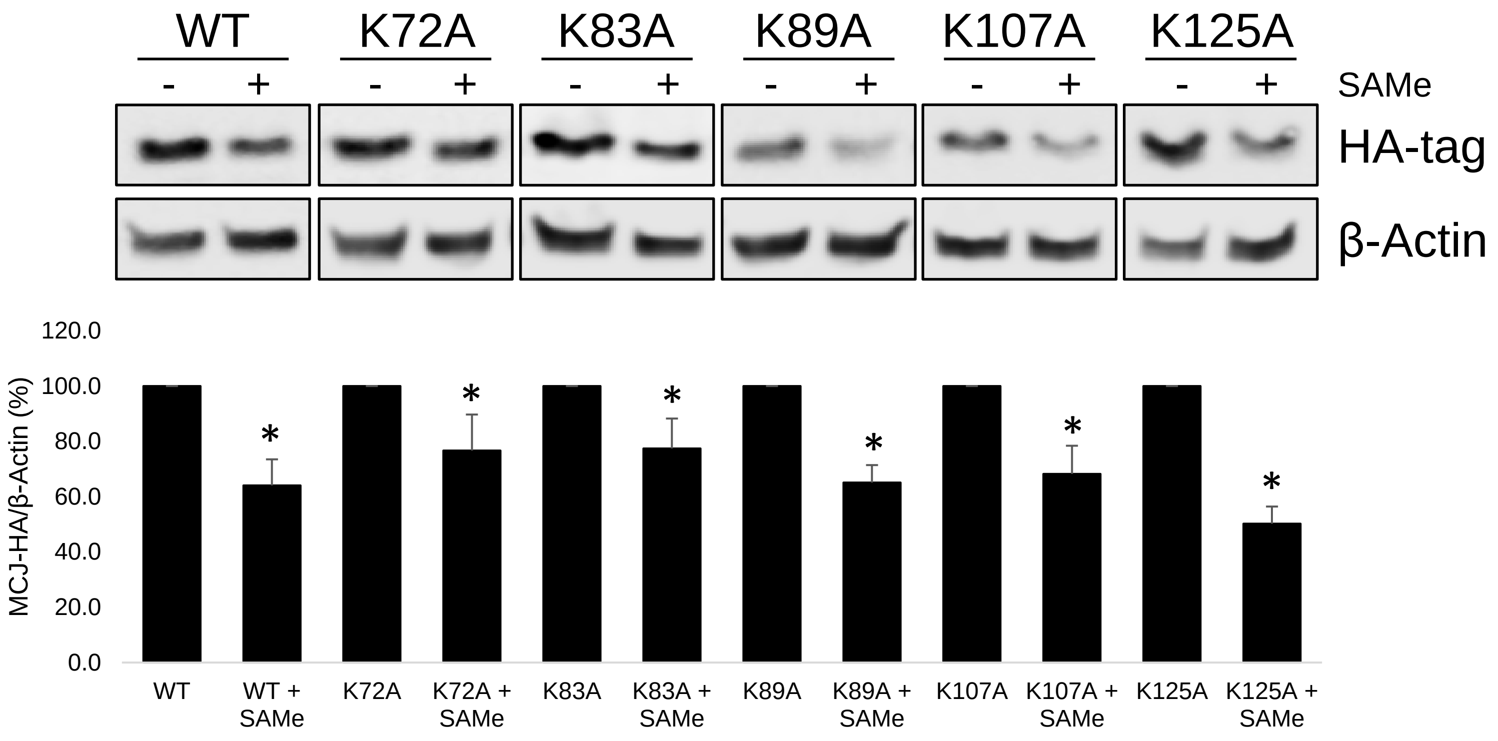

Figure S6

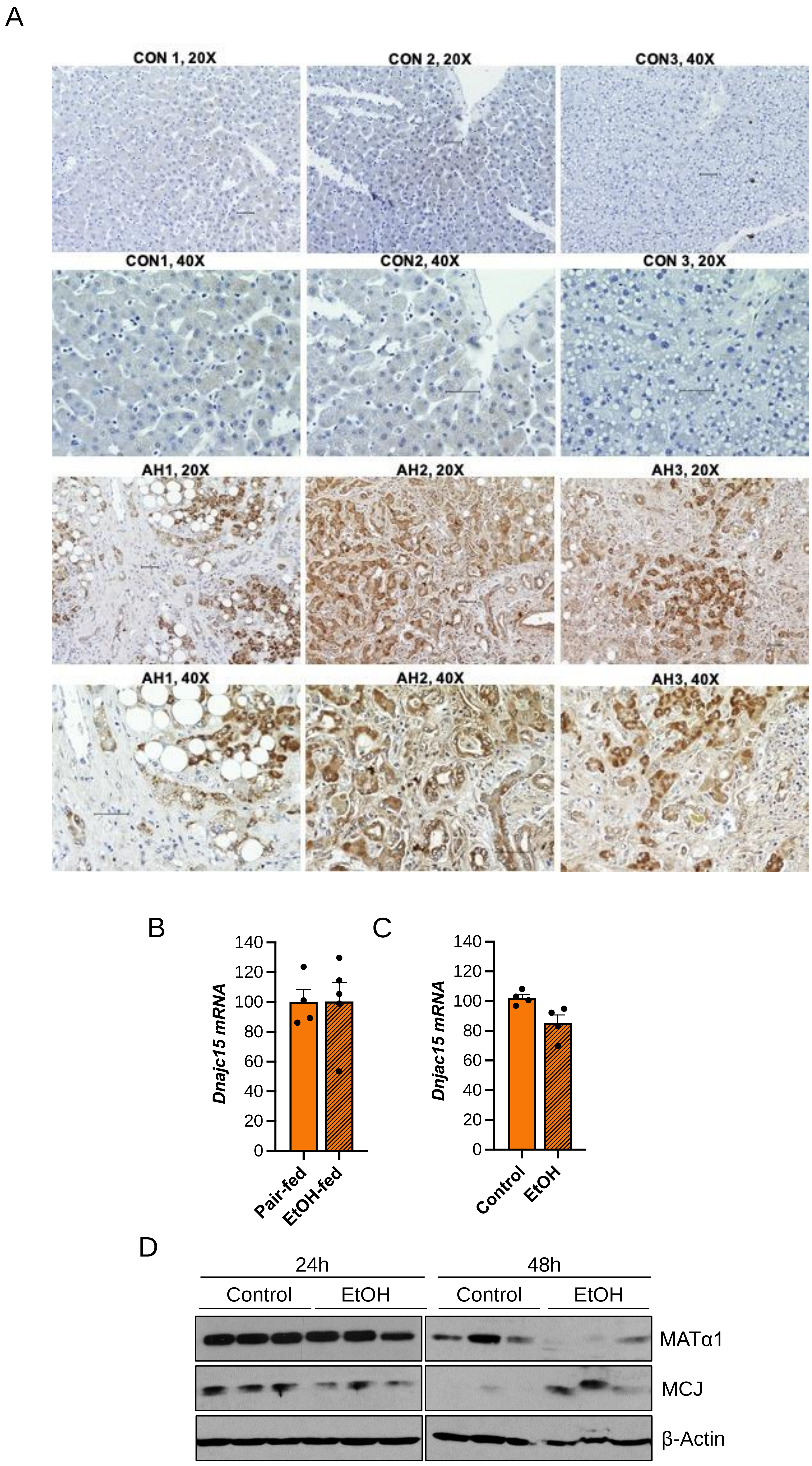
A
CON 1, 20X
CON 2, 20X
CON3, 40X
CON1, 40X
CON2, 40X
CON 3, 20X
AH1, 20X
AH2, 20X
AH3, 20X
AH1, 40X
AH2, 40X
AH3, 40X
B
Dnajc15 mRNA
0
20
40
60
80
100
120
140
Pair-fed
EtOH-fed
C
Dnjac15 mRNA
Control
EtOH
D
24h
48h
Control
EtOH
Control
EtOH
MATα1
MCJ
β-Actin

Figure S7

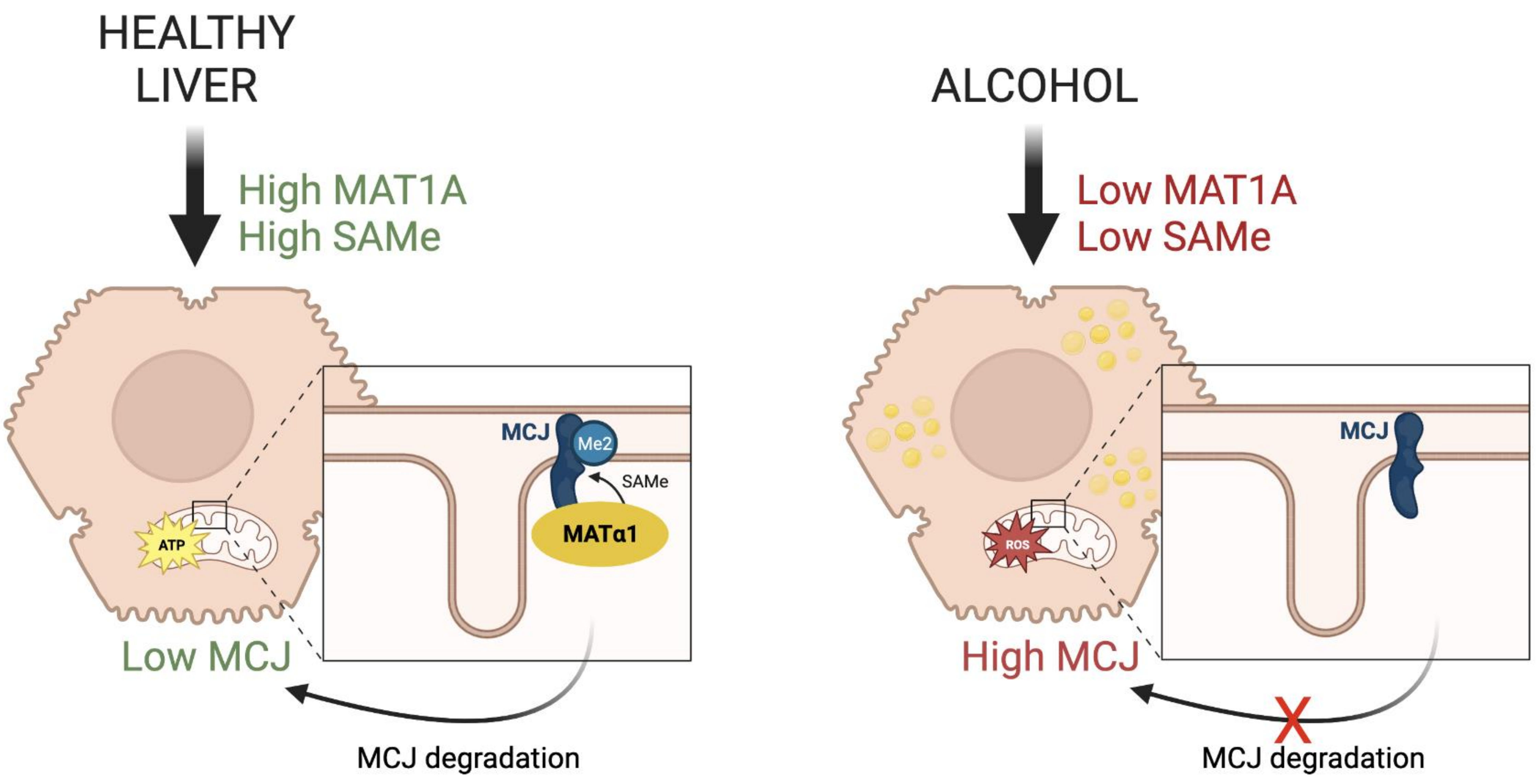

Graphical abstract
